# Critical role of thrombospondin-1 in promoting intestinal mucosal wound repair

**DOI:** 10.1172/jci.insight.180608

**Published:** 2024-07-30

**Authors:** Zachary S. Wilson, Arturo Raya-Sandino, Jael Miranda, Shuling Fan, Jennifer C. Brazil, Miguel Quiros, Vicky Garcia-Hernandez, Qingyang Liu, Chang H. Kim, Kurt D. Hankenson, Asma Nusrat, Charles A. Parkos

**Affiliations:** 1 Department of Pathology,; 2 Mary H. Weiser Food Allergy Center, and; 3 Department of Orthopedic Surgery, University of Michigan School of Medicine, Ann Arbor, Michigan, USA.

**Keywords:** Gastroenterology, Inflammation, Cell migration/adhesion, Cytoskeleton, Inflammatory bowel disease

## Abstract

Thrombospondin-1 (TSP1) is a matricellular protein associated with the regulation of cell migration through direct binding interactions with integrin proteins and by associating with other receptors known to regulate integrin function, including CD47 and CD36. We previously demonstrated that deletion of an epithelial TSP1 receptor, CD47, attenuates epithelial wound repair following intestinal mucosal injury. However, the mechanisms by which TSP1 contributes to intestinal mucosal repair remain poorly understood. Our results show upregulated TSP1 expression in colonic mucosal wounds and impaired intestinal mucosal wound healing in vivo upon intestinal epithelium–specific loss of TSP1 (*Villin*^Cre/+^ *Thbs1^fl/fl^* or *Thbs1*^ΔIEC^ mice). We report that exposure to exogenous TSP1 enhanced migration of intestinal epithelial cells in a CD47- and TGF-β1–dependent manner and that deficiency of TSP1 in primary murine colonic epithelial cells resulted in impaired wound healing. Mechanistically, TSP1 modulated epithelial actin cytoskeletal dynamics through suppression of RhoA activity, activation of Rho family small GTPase (Rac1), and changes in filamentous-actin bundling. Overall, TSP1 was found to regulate intestinal mucosal wound healing via CD47 and TGF-β1, coordinate integrin-containing cell–matrix adhesion dynamics, and remodel the actin cytoskeleton in migrating epithelial cells to enhance cell motility and promote wound repair.

## Abstract 2

We describe how the matricellular protein Thrombospondin-1 regulates focal adhesion turnover and actin cytoskeletal rearrangement to promote epithelial migration and drive mucosal repair.

## Graphical abstract

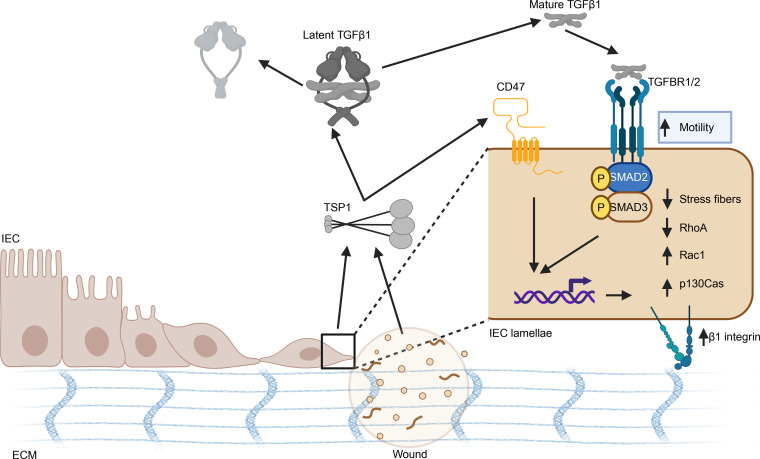

## Introduction

The intestinal epithelium forms a tight barrier that separates luminal contents from underlying tissues while remaining selectively permeable to allow the absorption of water and nutrients (1). It is thus not surprising that injury-induced disruption of the epithelial barrier is a characteristic feature of a number of chronic pathologic conditions of the intestine. For example, people with inflammatory bowel disease (IBD) are plagued with persistent intestinal epithelial damage and delayed repair, resulting in mucosal erosions/ulceration and exacerbation of disease symptoms. While therapeutic approaches have largely targeted the immune system in these diseases, there remains a critical unmet need for therapies aimed at restoring epithelial barrier function and promoting mucosal homeostasis.

In response to mucosal injury, epithelial cells migrate collectively as a sheet to cover injured or denuded regions in a process known as epithelial restitution (1). Collective epithelial migration is facilitated by coordinated remodeling of cell-cell and cell-matrix adhesions. Forward movement of the epithelium requires dynamic remodeling of integrin-containing focal adhesions and intracellular filamentous-actin–rich (F-actin–rich) protrusions at the leading edge that adhere to the extracellular matrix (ECM) (1). ECM proteins, such as collagen or fibronectin proteins, serve as structural components and act as signaling molecules that regulate cell-matrix interactions and cellular functions that intimately affect migration and proliferation. Matricellular proteins are a class of ECM proteins particularly well-known for promoting de-adhesive responses in cells such as endothelial cells (2). Thrombospondin-1 (TSP1; murine gene *Thbs1*) is a matricellular protein reported to play a role in regulating tissue remodeling and wound repair through binding interactions with integrin proteins and other receptors that control integrin function, including CD47 and CD36 (3–5). TSP1 has been shown to activate transforming growth factor-β1 (TGF-β1), leading to collagen deposition and re-epithelization of dermal wounds (6), but the role of TSP1 in regulating mucosal wound repair in the gut is poorly understood. Studies reporting TSP1 expression during intestinal inflammation highlight a potential significance of TSP1 in mediating repair and regeneration in the intestine (7). In particular, we recently demonstrated that CD47, an intestinal epithelially expressed receptor for TSP1, plays a critical role in regulating mucosal wound repair in vivo (8).

Given these observations, we performed studies to evaluate the role of TSP1 in models of intestinal mucosal injury and repair. We report that *Thbs1*-null mice have markedly impaired wound closure following biopsy-induced colonic injury. Experiments with bone marrow chimeras derived from TSP1-deficient mice indicated that both hematopoietic- and non–hematopoietic-derived TSP1 significantly contributed to mucosal repair. Furthermore, mice selectively deficient in intestinal epithelial cell–derived (IEC-derived) TSP1 had profound defects in repair of biopsy-induced mucosal wounds as well as impaired colonic mucosal healing following acute inflammatory injury with dextran sodium sulfate (DSS). In vitro studies using 2-dimensional (2D) primary cultures of human and mouse colonic epithelial cells demonstrated that TSP1 regulated actin cytoskeletal dynamics and focal adhesion formation in IECs in a TGF-β1–dependent manner with suppressed RhoA/myosin light chain (MLC) contractility and enhanced Rho family small GTPase (Rac1) signaling in migrating sheets of epithelial cells. These studies highlight for the first time to our knowledge how TSP1 guides migration of colonic epithelial cells following injury through regulation of focal adhesion turnover and Rac1-mediated actin cytoskeletal rearrangement.

## Results

### TSP1 expression is increased in colonic mucosa during wound repair.

**Figure 1:**
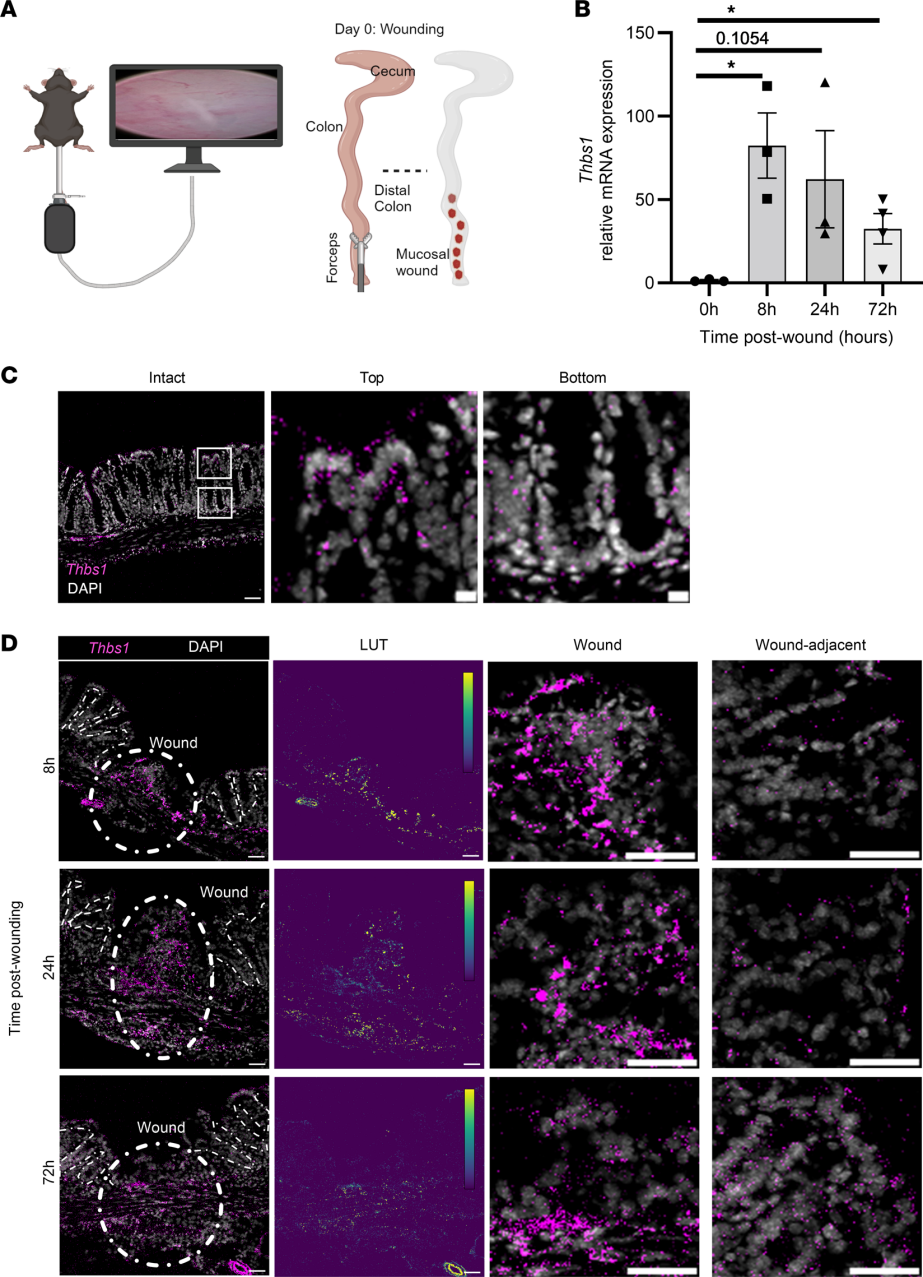
*Thbs1* is upregulated during biopsy-induced colonic mucosal wound repair. (**A**) Schematic of biopsy-based colonic mucosal wound repair assay. (**B**) Quantitative PCR (qPCR) of *Thbs1* mRNA after biopsy-based colonic mucosal wound repair at indicated time points (*n* = 3–4 mice). Performed 1-way ANOVA with Tukey multiple comparisons test. *P < 0.05. Mean ± SEM. (**C**) In situ hybridization (RNAscope) of *Thbs1* in intact mouse colon tissue (representative of *N* = 3 independent experiments). Scale bar = 50 μm, 10 μm insets. (**D**) In situ hybridization (RNAscope) of *Thbs1* in mouse colonic biopsy-based wounds at indicated time points (representative of *N* = 3 independent experiments). Lookup table (LUT) depicts the gradient of *Thbs1* expression. Scale bar = 50 μm. Insets shown on right for wound and wound-adjacent area.

To study spatiotemporal regulation of TSP1 expression during mucosal repair, levels of *Thbs1* mRNA were examined in intact colonic tissue and compared with colonic mucosal wounds at 8, 24, and 72 hours after biopsy-based wounding in mice using a miniaturized endoscopic technique (9) (Figure 1A). A significant increase in *Thbs1* mRNA expression was observed as early as 8 hours and persisted until 72 hours after injury relative to intact colonic tissue: 82.36-fold ± 19.63-fold at 8 hours (*P* = 0.0146), 62.19-fold ± 29.11-fold at 24 hours (*P* = 0.1054), and 32.42-fold ± 9.12-fold at 72 hours after wounding (*P* = 0.0351) (Figure 1B). Immunofluorescence analyses of intact murine colonic epithelium revealed increased expression of TSP1 that localized to the tops of colonic crypts relative to crypt bases (Figure 1C). After 8 hours *Thbs1* mRNA expression was observed in infiltrating immune cells within the wound (Figure 1D). Interestingly, an increase in *Thbs1* expression was also observed at the top of wound-adjacent epithelial crypts between 24 and 72 hours after injury (Figure 1D). These findings demonstrate important spatiotemporal and cell-specific regulation of TSP1 expression during colonic mucosal wound healing.

### TSP1 regulates intestinal wound healing in vivo.

**Figure 2:**
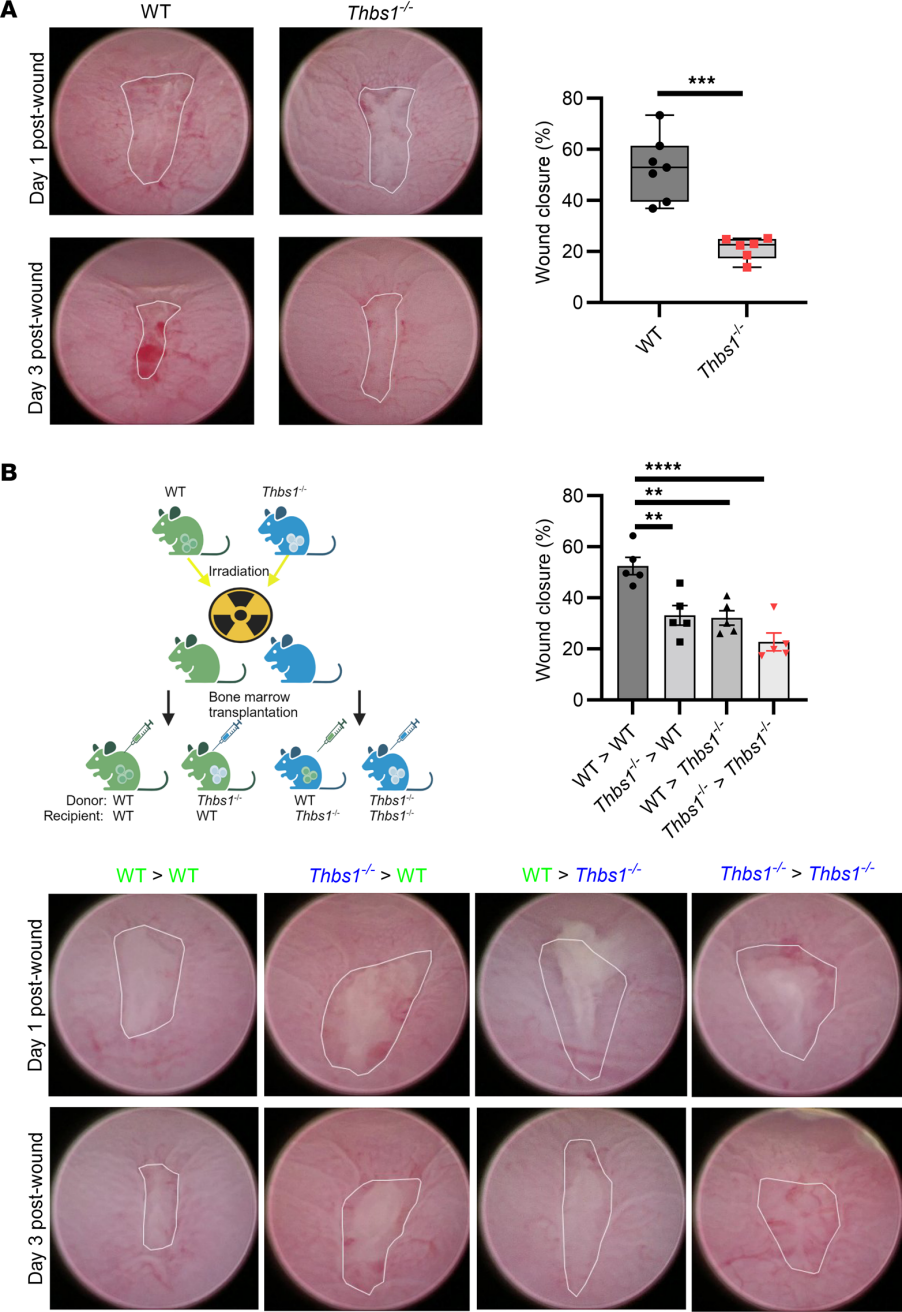
TSP1 is critical for murine colonic mucosal repair in both hematopoietic and nonhematopoietic cells. (**A**) In vivo wound closure following biopsy-based injury in WT and *Thbs1^–/–^* mice comparing day 1 and day 3 wounds (*n* = 7–8 mice each). Performed unpaired, 2-tailed Student’s *t* test. (**B**) In vivo wound closure following biopsy-based injury in bone marrow chimeric mice comparing day 1 and day 3 wounds (*n* = 5 mice each). Bone marrow chimeric mice are generated using 2 doses of 5 Gy separated by 4 hours in recipient mice and transplanting with bone marrow from WT and *Thbs1^–/–^* donor mice to generate all 4 possible combinations of donor > recipient: WT > WT, *Thbs1^–/–^* > WT, WT > *Thbs1^–/–^*, and *Thbs1^–/–^* > *Thbs1^–/–^*. Performed 1-way ANOVA with Tukey’s multiple comparisons test. Box-and-whisker plots show interquartile range, median (line), and minimum and maximum (whiskers). Histograms show mean ± SEM. **P < 0.01, ***P < 0.001, ****P < 0.0001.

The contribution of TSP1 to acute repair of colonic mucosal wounds was next examined. A significant decrease in wound closure in mice completely deficient in TSP1 (*Thbs1^–/–^*) relative to WT controls was observed (52.85% ± 4.73% in WT mice, 21.28% ± 1.77% in *Thbs1^–/–^* mice, *P* < 0.001) (Figure 2A). Since immune and nonimmune cells produce TSP1, we evaluated the relative contribution of these different cell types during mucosal wound repair in the colon using bone marrow chimeras. In such experiments, irradiated WT and *Thbs1^–/–^* mice were reconstituted with either WT or *Thbs1^–/–^* donor bone marrow (Figure 2B). Both hematopoietic-deficient (*Thbs1^–/–^* > WT) and non-hematopoietic–deficient (WT > *Thbs1^–/–^*) mice displayed statistically significant decreases in colonic wound closure relative to control (WT > WT) mice (Figure 2B). These data indicate that both hematopoietic (immune) and nonhematopoietic (nonimmune) cell–derived TSP1 play significant roles in regulating colonic wound repair.

### Epithelium-derived TSP1 plays an important role in regulating mucosal wound repair.

**Figure 3:**
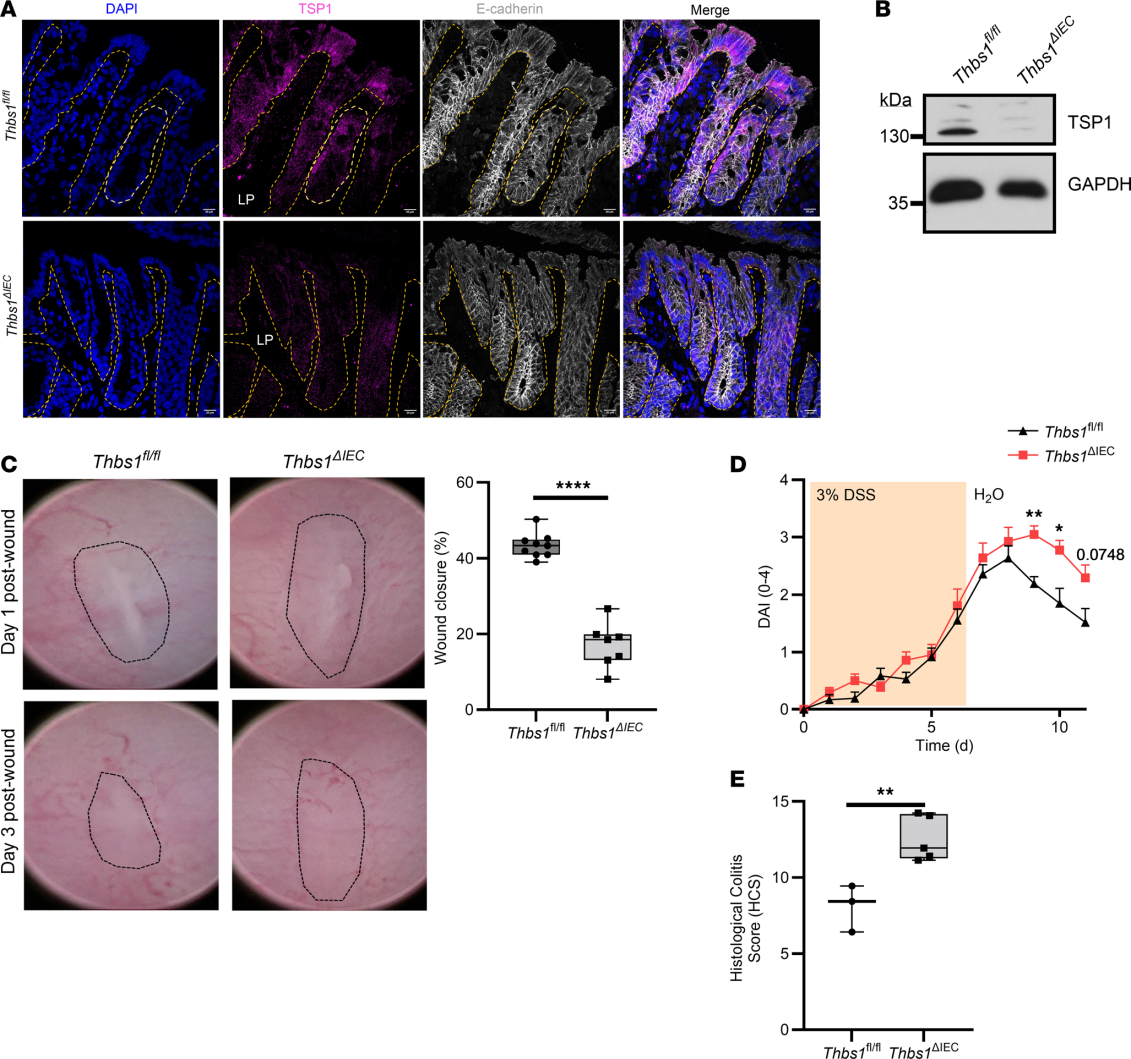
Epithelium-derived TSP1 is critical for in vivo mucosal wound repair. (**A**) Immunofluorescence of TSP1 in isolated murine colon tissue from epithelium-specific TSP1-knockout (*Thbs1*^ΔIEC^) and counterpart (*Thbs1^fl/fl^*) mice (representative of *N* = 3 independent experiments). Scale bar = 20 μm. LP, lamina propria. (**B**) Western blot of TSP1 in isolated murine colon colonoids from *Thbs1*^ΔIEC^ and *Thbs1^fl/fl^* (representative of *n* = 3 mice). (**C**) In vivo wound closure following biopsy-based injury in *Thbs1*^ΔIEC^ and *Thbs1^fl/fl^* mice comparing day 1 and day 3 wounds (*n* = 7–8 mice each). Performed unpaired, 2-tailed Student’s *t* test. (**D**) DSS-induced colitis in *Thbs1*^ΔIEC^ and *Thbs1^fl/fl^* mice showing disease activity index (DAI) (*n* = 12–14 mice each). Performed 2-way ANOVA with Holm-Šídák multiple-comparison test. Mean ± SEM. (**E**) Histological colitis score (HCS) of *Thbs1*^ΔIEC^ and *Thbs1^fl/fl^* mice 9 days after DSS-induced colitis (*n* = 3–5 mice each). Performed unpaired, 2-tailed Student’s *t* test. Box plots show interquartile range, median (line), and minimum and maximum (whiskers). *P < 0.05, **P < 0.01, ***P < 0.001, ****P < 0.0001.

Given the observed increase in expression of TSP1 in wound-adjacent IECs and the well-appreciated importance of nonimmune cell–derived TSP1 in repair responses, we evaluated the contribution of epithelially expressed TSP1 to intestinal mucosal wound repair. Selective depletion of TSP1 in IECs from *Villin*^Cre/+^ *Thbs1^fl/fl^* or *Thbs1*^ΔIEC^ mice was verified by immunofluorescence and immunoblotting (Figure 3, A and B). There was significantly decreased colonic wound closure in *Thbs1*^ΔIEC^ mice compared with *Thbs1^fl/fl^* mice (43.34% ± 1.08% in *Thbs1^fl/fl^* mice, 17.11% ± 2.25% in *Thbs1*^ΔIEC^ mice, *P* < 0.0001) (Figure 3C), indicating that epithelial expression of TSP1 is necessary for mucosal repair in the colon. To determine the importance of IEC-derived TSP1 during repair following inflammatory injury in the colon, *Thbs1^fl/fl^* and *Thbs1*^ΔIEC^ mice were subjected to DSS colitis and monitored during recovery. Importantly, *Thbs1*^ΔIEC^ mice exhibited delayed recovery as reflected by increased disease activity index (DAI) scores on days 9 and 10 (Figure 3D). Histological analyses of colonic tissue isolated on day 9 revealed increased inflammatory injury, as determined by measures of inflammation and ulceration, in *Thbs1*^ΔIEC^ mice compared with control mice (Figure 3E). Taken together, these data indicate that epithelially derived TSP1 plays an important role in driving intestinal epithelial wound repair in vivo.

### Exogenously added TSP1 also promotes epithelial wound closure.

**Figure 4:**
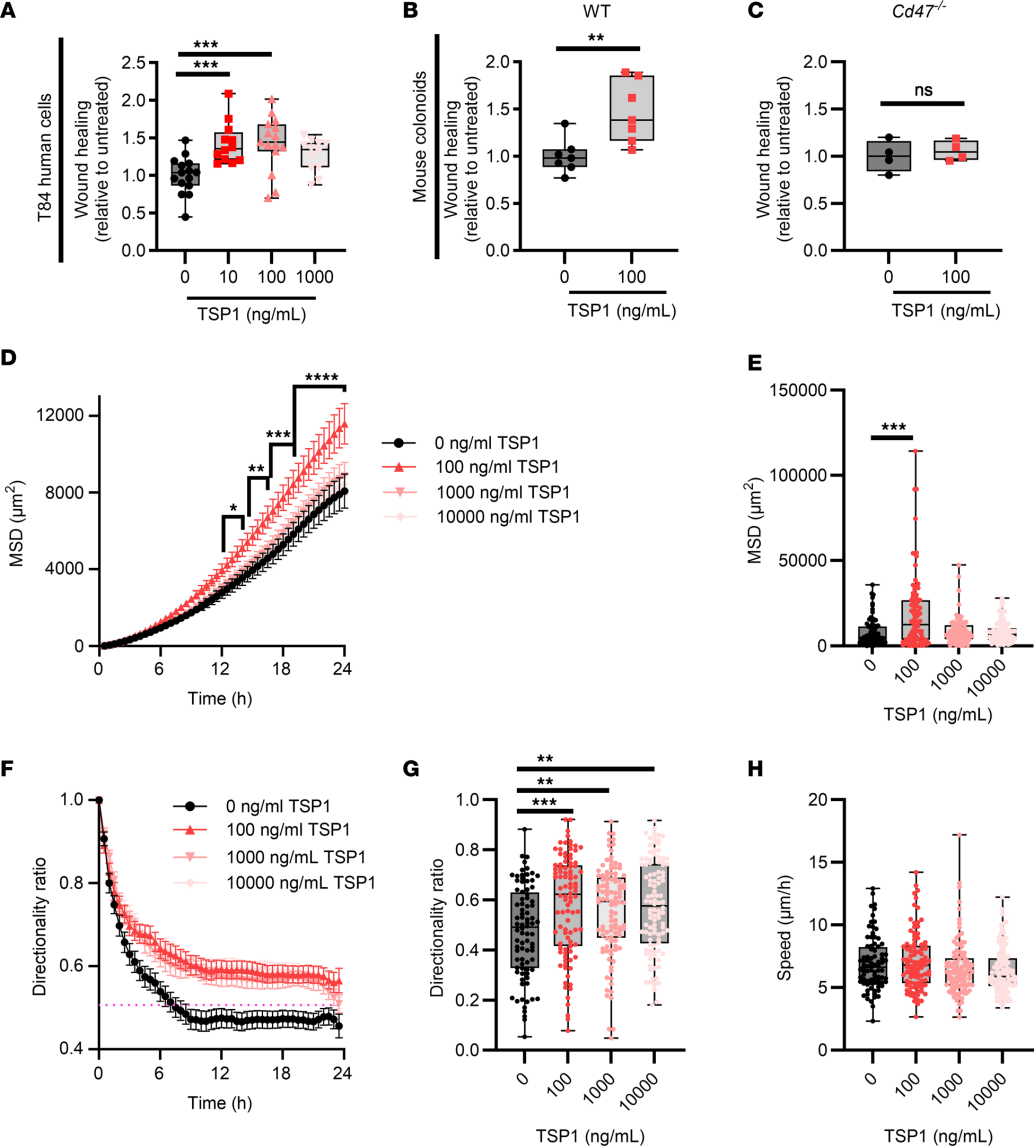
IEC migration is enhanced in response to TSP1. (**A**) Migration following scratch wounding for T84 human cells in response to indicated concentrations of TSP1 after 24 hours (*n* = 12–15, *N* = 3 independent experiments). Performed 1-way ANOVA with Tukey’s multiple comparisons test. (**B**) Migration following scratch wounding for WT mouse IECs in response to TSP1 after 24 hours (*n* = 6, *N* = 3 independent experiments). Performed unpaired, 2-tailed Student’s *t* test. (**C**) Migration following scratch wounding for *Cd47^–/–^* mouse IECs in response to TSP1 after 24 hours (*n* = 4, *N* = 3 independent experiments). Performed unpaired, 2-tailed Student’s *t* test. (**D**) Mean squared displacement (MSD) measurements of migrating WT mouse IEC monolayers in response to indicated concentrations of TSP1 over 24 hours (*n* > 80 per group, *N* = 3 independent experiments). (**E**) Quantification of MSD 24 hours after treatment with indicated concentrations of TSP1 (*n* > 80 per group, *N* = 3 independent experiments). Performed Kruskal-Wallis 1-way ANOVA with Dunn’s multiple comparisons test. (**F**) Directionality ratio measurements of migrating WT mouse IEC monolayers in response to indicated concentrations of TSP1 over 24 hours (*n* > 80 per group, *N* = 3 independent experiments). (**G**) Quantification of directionality ratio 24 hours after treatment with indicated concentrations of TSP1 (*n* > 80 per group, *N* = 3 independent experiments). Performed Kruskal-Wallis 1-way ANOVA with Dunn’s multiple comparisons test. (**H**) Quantification of average speed 24 hours after treatment with indicated concentrations of TSP1 (*n* > 80 per group, *N* = 3 independent experiments). Performed Kruskal-Wallis 1-way ANOVA with Dunn’s multiple comparisons test. Box plots show interquartile range, median (line), and minimum and maximum (whiskers). Histograms show mean ± SEM. *P < 0.05, **P < 0.01, ***P < 0.001, ****P < 0.0001.

To further probe how TSP1 promotes re-epithelization and repair of mucosal wounds, in vitro dose-response studies in which TSP1 was added to cultures of scratch-wounded epithelial monolayers followed by assessment of wound closure were performed. Confluent monolayers of T84 IECs were scratch-wounded and incubated with physiologically relevant concentrations of full-length recombinant human TSP1 (10). A significant increase in IEC wound closure was observed following exposure to 10 and 100 ng/mL (but not 1 μg/mL) TSP1 — 1.428-fold ± 0.08-fold with 10 ng/mL (*P* = 0.001), 1.425-fold ± 0.095-fold with 100 ng/mL (*P* = 0.0005), 1.273-fold ± 0.062-fold with 1 μg/mL (*P* = 0.0676) — suggesting a window of optimal concentration range(s) at which TSP1 acts to enhance wound closure (Figure 4A). Similar to effects observed for human IECs, addition of exogenous murine TSP1 at a concentration of 100 ng/mL resulted in enhanced wound closure in monolayers of colonoid-derived murine IECs (Figure 4B). Given that CD47, a well-appreciated epithelial receptor for TSP1, has been shown to play an important role in regulating mucosal wound healing (8), we assessed whether pro-repair effects of TSP1 are altered in IECs lacking CD47 expression. Importantly, wound repair was unchanged in TSP1-treated *Cd47^–/–^* IECs, suggesting that TSP1-mediated effects on IEC wound repair are, in part, dependent on the intestinal epithelial TSP1 receptor CD47 (Figure 4C and Supplemental Figure 1; supplemental material available online with this article; https://doi.org/10.1172/jci.insight.180608DS1).

During wound healing, epithelial cells undergo collective cell migration with leader cells generating traction forces that act to pull follower cells along a collective trajectory (11). To measure TSP1-driven collective migration in vitro, leader cells along the edges of migrating murine IEC monolayers were tracked over 24 hours and mean squared displacement (MSD) (a robust measurement of the directness and speed of cells) were calculated (12). As can be seen in Figure 4, D and E, 100 ng/mL TSP1 significantly increased MSD of murine IECs at time points between 13 and 24 hours after wounding. In contrast, MSD was unchanged following treatment with 1,000 and 10,000 ng/mL TSP1, once again highlighting dose-dependent functional effects of TSP1 (Figure 4, D and E). As 100 ng/mL TSP1 was found to consistently promote epithelial migration, this concentration was used for all subsequent experiments. Interestingly, the directionality ratio (or straight-line motion of a cell) was increased by TSP1, suggesting induction of a more linear trajectory (Figure 4, F and G). However, the average speed of migration was not increased by TSP1 (Figure 4H), suggesting that TSP1 alters directionality and not the overall speed of migrating IECs.

### Exogenously added TSP1 promotes focal adhesion formation.

**Figure 5:**
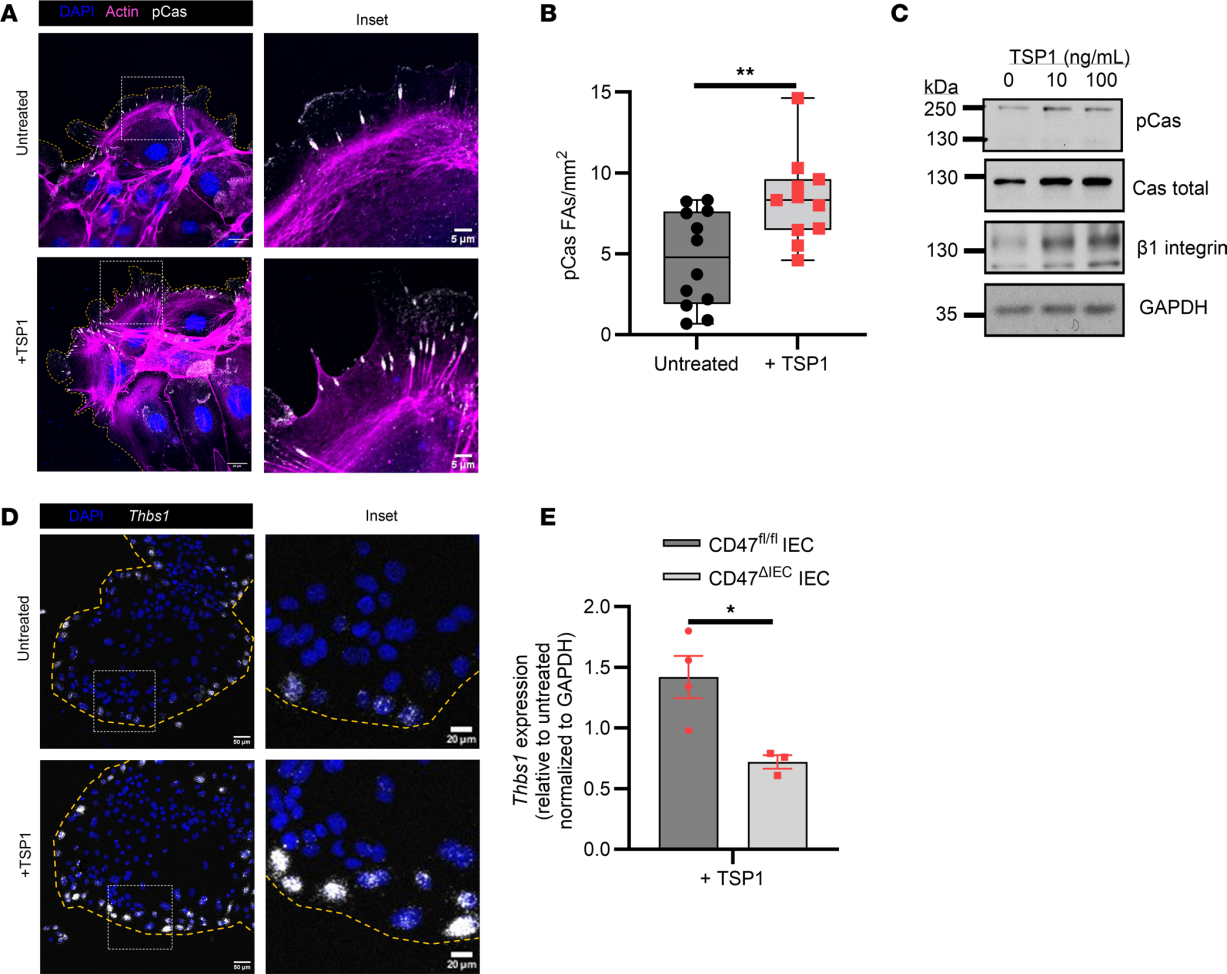
TSP1 enhances IEC migration with enhanced focal adhesion formations at the periphery of leading cells. (**A**) Immunofluorescence of p130Cas pY410 (pCas) and actin at the leading cells of migrating WT mouse IEC monolayers following 8 hours of TSP1 treatment (*N* = 3 independent experiments). Scale bar = 20 μm, 5 μm inset. (**B**) Quantification of p130Cas pY410 (pCas) focal adhesions. Performed unpaired, 2-tailed Student’s *t* test. (**C**) Western blot of phosphorylated p130Cas Y410, total p130Cas, β1 integrin, and GAPDH in response to indicated TSP1 treatment at 24 hours after wounding (representative of *N* = 3 independent experiments). (**D**) In situ hybridization (RNAscope) of *Thbs1* at the leading edge of migrating monolayers of WT mouse IECs in response to 4 hours TSP1 treatment (representative of *N* = 3 independent experiments). Scale bar = 50 μm, 20 μm inset. (**E**) qPCR of thrombospondin-1 mRNA (*Thbs1*) in CD47-replete and deplete IECs in response to TSP1 treatment 24 hours after wounding (*n* = 3–4 mice). Performed unpaired, 2-tailed Student’s *t* test. Box plots show interquartile range, median (line), and minimum and maximum (whiskers). Histograms show mean ± SEM. *P < 0.05, **P < 0.01.

Previous studies have shown that epithelial migration is a complex multistep process dependent on integrin β1–mediated focal adhesion formation, phosphorylation of adaptor proteins such as p130Cas, dynamic rearrangement of the actin cytoskeleton, and lamellipodia stabilization (13–16). We thus performed immunofluorescence labeling of phosphorylated p130Cas Y410 (pCas) and F-actin in migrating IECs exposed to exogenously added TSP1 and observed increased activation of p130Cas in lamellipodial processes (Figure 5, A and B). Furthermore, immunoblotting analyses of wounded murine epithelial monolayers revealed increased levels of pCas, total p130Cas (Cas), and integrin β1 in response to TSP1 (Figure 5C). Next, we performed an adhesion array assay to measure attachment of IECs to various ECM substrates following TSP1 treatment. Interestingly, we observed an increase in attachment of IECs to fibronectin, but not to other substrates, including collagen I and IV (Supplemental Figure 2). Taken together, these findings suggest that TSP1 in the extracellular milieu plays an important role in IEC migration by stimulating increased formation of focal adhesions and promotion of adhesion to substrates including fibronectin.

As TSP1 has been reported to stimulate formation of cytoskeletal attachments within lamellipodia that facilitate migration (17), and we observed increased TSP1 expression by epithelial cells during wound healing (Figure 1D), we performed experiments using in situ hybridization to spatially visualize *Thbs1* RNA expression in collectively migrating murine IECs. Specific expression of *Thbs1* mRNA was observed in leader cells of migrating IEC sheets, with increased expression observed upon exposure to exogenously added TSP1 (Figure 5D), raising the possibility that endogenous TSP1 production is enhanced in the presence of exogenously added TSP1, perhaps as part of an autocrine signaling feedback loop. Interestingly, upregulation of endogenous *Thbs1* mRNA by exogenously added TSP1 was not observed in *Cd47^–/–^* IECs, suggesting dependence of these effects on the TSP1 receptor CD47 (Figure 5E). Collectively, these findings suggest that TSP1-driven migration of IECs is mediated by enhanced focal adhesion formation linked to an IEC-maintained self-gradient of TSP1.

### TGF-β1 mediates TSP1-dependent effects on IEC migration.

**Figure 6:**
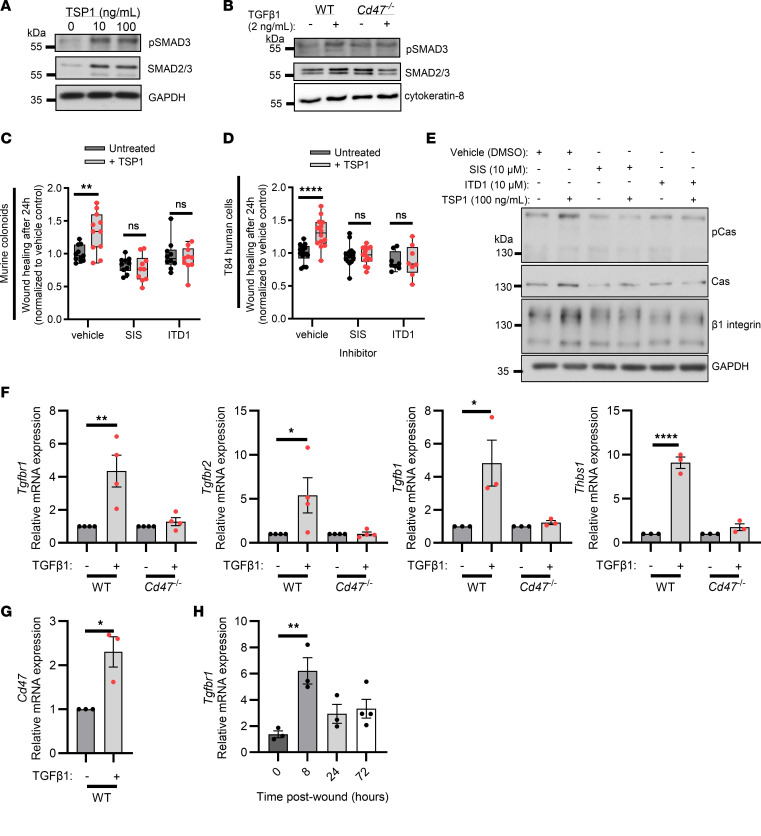
IEC migration in response to TSP1 is TGF-β1 dependent. (**A**) Western blot of wounded murine IECs treated with TSP1 for 24 hours at the indicated concentration probed for phosphorylated SMAD3 S423/425, SMAD2/3, and GAPDH (representative of *N* = 3 independent experiments). (**B**) Western blot of WT and *Cd47*^–*/*–^ wounded murine IECs treated with TGF-β1 (2 ng/mL) for 24 hours probed for phosphorylated SMAD3 S423/425, SMAD2/3, and cytokeratin-8 (representative of *N* = 4 independent experiments). (**C**) Wound closure rates at 24 hours after wounding in a scratch wound assay of murine IECs treated with and without TSP1, SMAD3 inhibitor SIS3, and TGF-β1 inhibitor ITD1. Performed 2-way ANOVA with Holm-Šídák multiple-comparison test. (**D**) Wound closure rates at 24 hours after wounding in a scratch wound assay of T84 IECs treated with and without TSP1, SIS3, and ITD1. Performed 2-way ANOVA with Holm-Šídák multiple-comparison test. (**E**) Western blot of wounded murine IECs treated for 24 hours with and without TSP1, SIS3, and ITD1 (representative of *N* = 2 independent experiments). (**F**) qPCR of mRNA from TGF-β1–related genes *Tgfbr1*, *Tgfbr2*, *Tgfb1*, and *Thbs1* in WT and *Cd47^–/–^* murine IECs treated with TGF-β1 (2 ng/mL) 4 hours after wounding (*N* = 3–4 independent experiments). Performed 1-way ANOVA with Tukey’s multiple comparison test. (**G**) qPCR of *Cd47* mRNA in WT murine IECs treated with TGF-β1 (2 ng/mL) 4 hours after wounding (*N* = 3 independent experiments). Performed unpaired, 2-tailed Student’s *t* test. (**H**) qPCR of *Tgfbr1* mRNA after biopsy-based colonic mucosal wound repair at indicated time points (*n* = 3–4 mice). Performed 1-way ANOVA with Tukey’s multiple-comparison test. Box plots show interquartile range, median (line), and minimum and maximum (whiskers). Histograms show mean ± SEM. *P < 0.05, **P < 0.01, ****P < 0.0001.

TSP1 has been shown to be an important activator of TGF-β1 in the context of skin wounds (18). We thus investigated TGF-β1 signaling in wounded and TSP1-treated colonoids to elucidate a potential mechanism by which TSP1 promotes colonic epithelial repair. SMAD3 is an essential signaling protein downstream of TGF-βR1/2, which is part of the canonical SMAD complex that traffics to the nucleus and promotes transcription of TGF-β1–stimulated genes (19). Immunoblotting analyses revealed increased levels of phosphorylated SMAD3 and total SMAD2/3 protein in wounded murine IECs upon TSP1 treatment, suggesting TSP1-mediated activation of TGF-β1 signaling in migrating IECs (Figure 6A). To determine whether TGF-β1 activation downstream of TSP1 is regulated by CD47, SMAD3 phosphorylation was measured in wounded WT and CD47-deficient colonoids. As can be seen in Figure 6B, TGF-β1–mediated increases in phosphorylated SMAD3 compared with total SMAD2/3 protein were not observed in wounded CD47-deficient IEC monolayers (Figure 6B and Supplemental Figure 3). Together, these data suggest that TSP1 in IECs signals by activation of TGF-β1 and that these responses are dependent on the receptor CD47.

To verify that enhanced wound healing responses mediated by TSP1 were dependent on TGF-β1 signaling, we measured the rate of wound closure in TSP1-treated wounded murine colonoids exposed to TGF-β1 inhibitors SIS3 and ITD1 (20, 21). As expected, TSP1 alone increased the rate of wound closure relative to control untreated at 24 hours after wounding (Figure 6C). However, treatment with TGF-β1 inhibitors reversed positive effects of TSP1 on IEC wound closure (Figure 6C). Effects of TSP1-driven TGF-β1 signaling during wound closure were further verified using another IEC model consisting of wounded T84 human epithelial monolayers. As was seen for murine IECs, inhibition of TGF-β1 signaling prevented TSP1-mediated increases in wound healing for human IECs (Figure 6D). Further, immunoblotting analyses revealed that TSP1-mediated increases in pCas, Cas, and β1 integrin were attenuated by both TGF-β1 inhibitors SIS3 and ITD1 (Figure 6E), suggesting that the enhanced focal adhesion formation seen after TSP1 treatment is dependent on TGF-β1 signaling.

Given that autocrine TGF-β1 function in cancer is regulated through positive and negative feedback signaling networks (22), we examined expression of key proximal TGF-β1 signaling receptor components in wounded murine colonoids exposed to TGF-β1. In response to TGF-β1, wounded WT IECs upregulated *Tgfbr1*, *Tgfbr2*, *Tgfb1*, *Thbs1*, and *Cd47* mRNA expression (Figure 6, F and G). In contrast, CD47-deficient IECs did not upregulate mRNA expression in response to TGF-β1, though the baseline expression was elevated in vitro relative to WT (Figure 6F and Supplemental Figure 4). TSP1 treatment was similar to TGF-β1 treatment in upregulation of TGF-β1 signaling receptor components (Supplemental Figure 5). Taken together, Figure 6, F and G, suggest a feedback loop where TGF-β1 promotes signaling by TSP1 through further activation of TGF-β1 and CD47 to drive mucosal wound repair. In keeping with in vitro findings, elevated expression of *Tgfbr1* was observed in colonic mucosal wounds 8 hours following injury with elevated levels of expression persisting for 3 days (Figure 6H). These data highlight induction of TGF-βR1 expression following IEC wound healing that would facilitate increased capacity for TGF-β1–mediated signaling during repair. Together, these data suggest that in IECs, TSP1 signals through TGF-β1 to promote wound repair though a positive feedback loop involving TSP1 and TGF-β1.

### Delayed wound repair responses in TSP1-deficient IECs can be rescued by exogenous TSP1.

**Figure 7:**
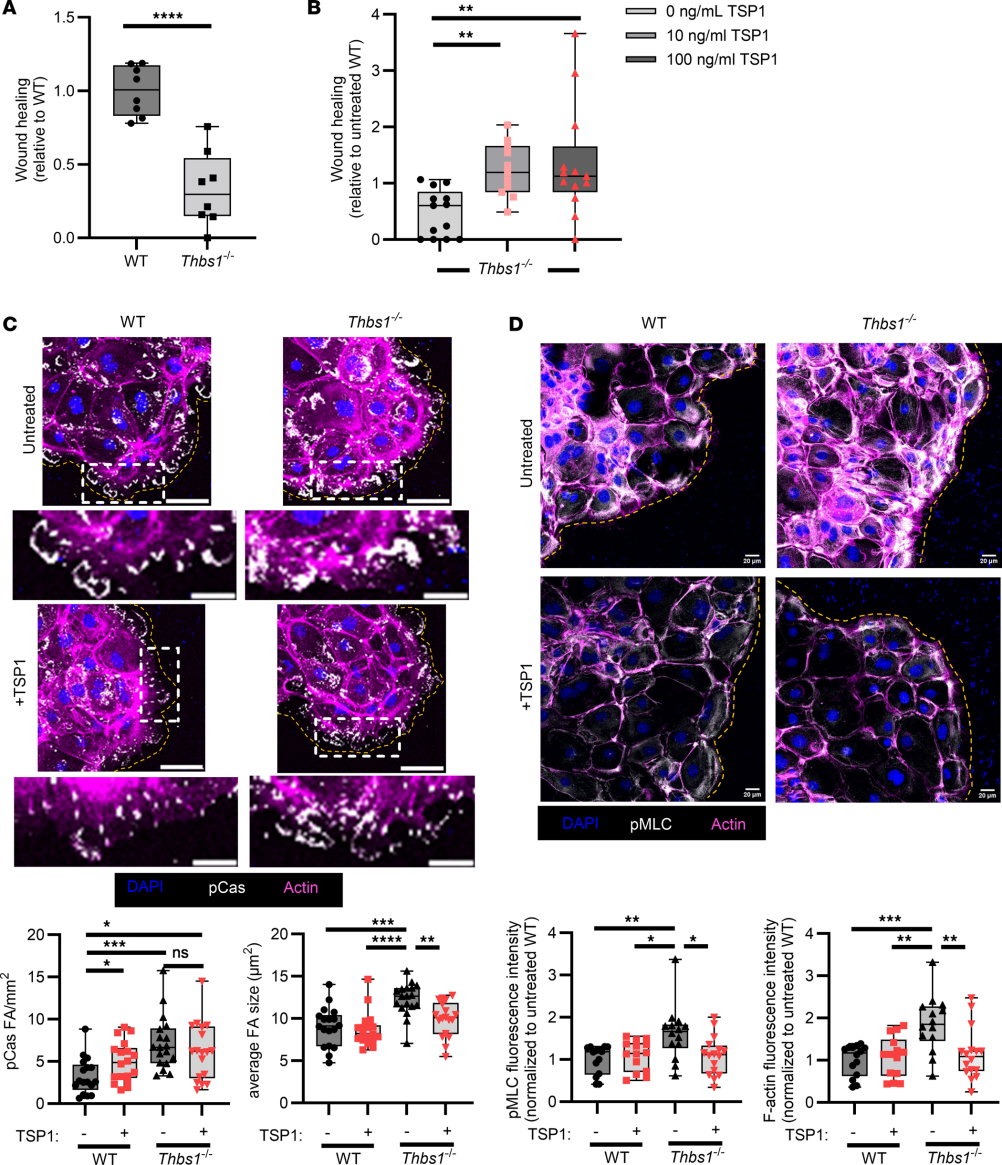
Deficiency in TSP1 impairs epithelial repair and cytoskeletal rearrangement. (**A**) Wound closure rates at 20 hours after wounding in a scratch wound assay of WT and *Thbs1^–/–^* murine IECs (*n* = 8, *N* = 3 independent experiments). Performed unpaired, 2-tailed Student’s *t* test. (**B**) Wound closure rates at 24 hours after wounding in a scratch wound assay of *Thbs1^–/–^* murine IECs in response to TSP1 (*n* = 13, *N* = 3 independent experiments). Performed Kruskal-Wallis 1-way ANOVA with Dunn’s multiple comparisons test. (**C**) Immunofluorescence of phosphorylated p130Cas Y410 and actin in WT and *Thbs1^–/–^* murine IECs in response to 24-hour treatment with TSP1 (representative of *N* = 3 experiments). Scale bar = 50 μm, 20 μm for inset. Performed 1-way ANOVA with Tukey’s multiple-comparison test. (**D**) Immunofluorescence of pMLC S19 and actin in WT and *Thbs1^–/–^* murine IECs in response to 24-hour treatment with TSP1 (representative of *N* = 3 experiments). Scale bar = 20 μm. Quantification of immunofluorescence below. Performed 1-way ANOVA with Tukey’s multiple-comparison test. Box plots show interquartile range, median (line), and minimum and maximum (whiskers). *P < 0.05, **P < 0.01, ***P < 0.001, ****P < 0.0001.

As shown in Figure 3C, endogenously produced TSP1 in IECs is an important regulator of colonic mucosal wound repair in vivo. To better understand the role of epithelially derived TSP1 during repair in vitro, we isolated WT and TSP1-deficient colonoids from mice and performed scratch-wounding assays. Wound closure in TSP1-deficient IECs was significantly decreased compared with TSP1-expressing IECs (Figure 7A, *P* < 0.0001), suggesting that the decreased wound repair observed in Figure 3C is related to a reduced migratory capacity of TSP1-deficient IECs. Importantly, exogenous addition of 10 and 100 ng/mL recombinant full-length TSP1 enhanced wound closure of *Thbs1^–/–^* IEC monolayers (Figure 7B), demonstrating that addition of TSP1 can rescue functional effects observed with TSP1-deficient IECs in vitro. Focal adhesion formation and F-actin bundling in *Thbs1^–/–^* IECs were evaluated to gain insight into molecular events responsible for the observed defects in migration. Surprisingly, we found that migrating *Thbs1^–/–^* IEC monolayers had increased levels of pCas-containing focal adhesions and F-actin relative to WT IEC monolayers (Figure 7C). Furthermore, addition of exogenous TSP1 reduced F-actin bundling and pCas-containing focal adhesion size in Thbs1^–/–^ IECs (Figure 7C). Therefore, the decreased pCas signal observed with exogenously added TSP1 suggests that TSP1 may play a role in breakdown of focal adhesions and thus promote focal adhesion turnover. Excessive stress fiber and focal adhesion formation in the appropriate context have been found to be deleterious to cell motility (23–25). This is exemplified in leukocytes, which are generally known for fast motility with low stress fiber and focal adhesion formation (23, 26). Given elevated F-actin bundling observed in *Thbs1^–/–^* IECs, we examined activation of MLC to determine if TSP1 is altering cellular contractility and the formation of actin stress fibers (27, 28). Analysis of phosphorylation of MLC S19 (pMLC) by immunofluorescence revealed elevated activated pMLC as well as increased F-actin bundling in migrating *Thbs1^–/–^* IEC monolayers (Figure 7D). Levels of active pMLC and F-actin bundling were greatly decreased after TSP1 treatment with localization of pMLC in cells at the leading edge of migrating monolayers (Figure 7D). These data suggest that, in addition to promoting focal adhesion formation, TSP1 also regulates establishment of focal adhesions and F-actin bundling during IEC migration.

### TSP1 regulates actin cytoskeletal dynamics through Rho GTPases RhoA and Rac1.

**Figure 8:**
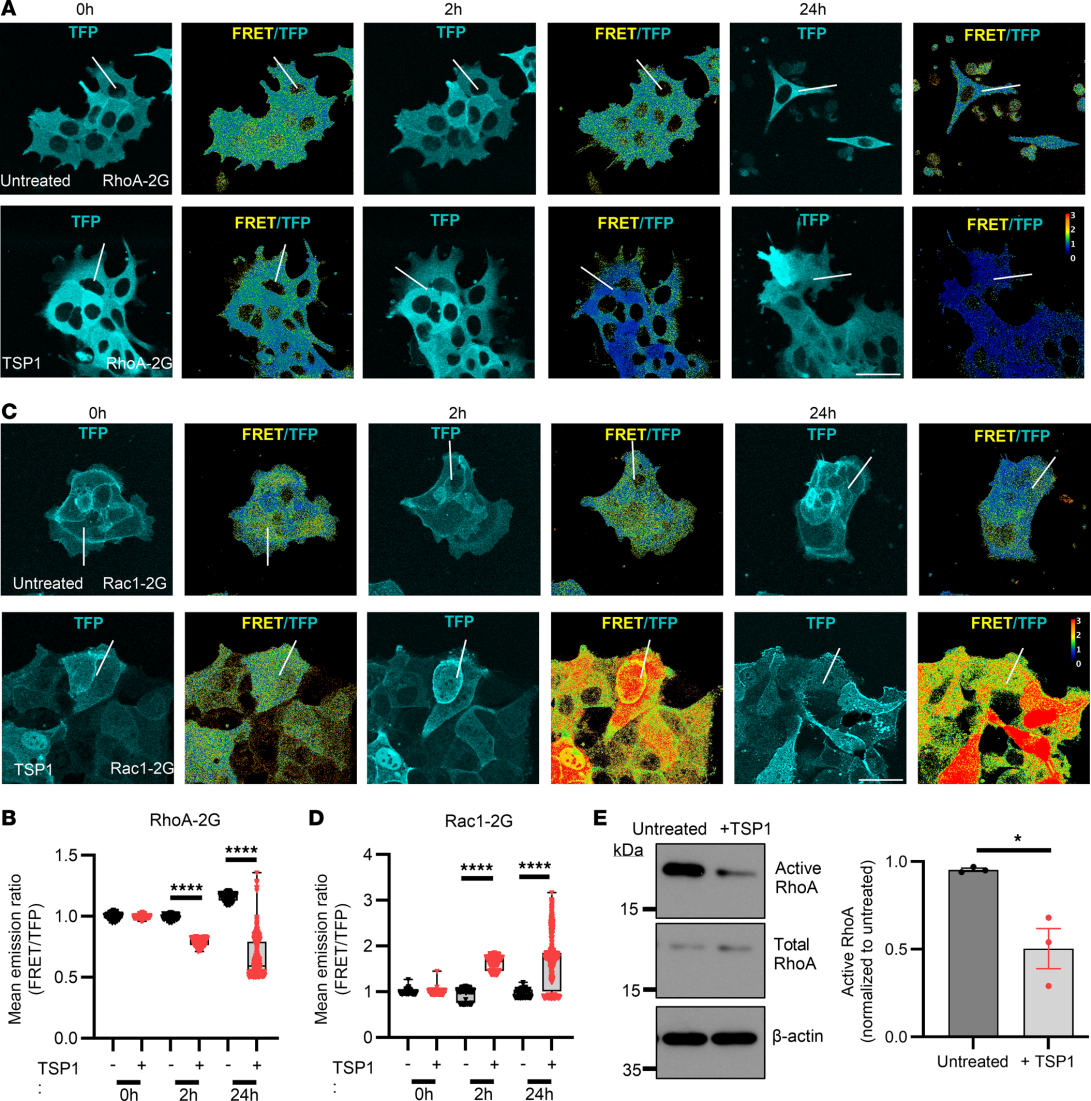
TSP1 promotes Rac1 and inhibits RhoA activity. (**A**) Immunofluorescence of FRET and total fluorescence in migrating SKCO15 cells expressing RhoA-2G construct (representative of *N* = 3 independent experiments). (**B**) Quantification of FRET/teal fluorescent protein (TFP) mean emission ratio in SKCO15 cells expressing RhoA-2G construct (*N* = 3 independent experiments). Performed Kruskal-Wallis 1-way ANOVA with Dunn’s multiple comparisons test. (**C**) Immunofluorescence of FRET and total fluorescence in migrating SKCO15 cells expressing Rac1-2G construct (representative of *N* = 3 independent experiments). (**D**) Quantification of FRET/TFP mean emission ratio in SKCO15 cells expressing Rac1-2G construct (*N* = 3 independent experiments). Performed Kruskal-Wallis 1-way ANOVA with Dunn’s multiple comparisons test. (**E**) Western blot of GTP-bound RhoA (active RhoA), total RhoA, and β-actin in wounded SKCO15 cells with and without treatment of 100 ng/mL TSP1 for 24 hours (*N* = 3 experiments). Performed unpaired, 2-tailed Student’s *t* test. Box plots show interquartile range, median (line), and minimum and maximum (whiskers). Histograms show mean ± SEM. *P < 0.05, ****P < 0.0001.

Rho GTPases are molecular switches that control downstream signal transduction networks and play a pivotal role in regulating actin cytoskeletal migratory dynamics (29). Therefore, to further determine effects of TSP1 on actin cytoskeletal assembly, the activation of Rho GTPases Rac1 and RhoA was evaluated following treatment of IECs with exogenously added TSP1. SKCO15 cells (a human IEC line) expressing a RhoA FRET biosensor (30) were used to determine spatiotemporal activation of RhoA, a GTPase best characterized as regulating actomyosin contractility and stress fiber formation (29). As shown in Figure 8, A and B, addition of TSP1 resulted in decreased RhoA activation in IECs at time points between 2 and 24 hours, which correlated with decreased pMLC as observed in Figure 7D, suggesting that actomyosin contractility is suppressed in IECs following exposure to TSP1. Reduced actomyosin contractility with reduced stress fiber formation would normally suggest lower traction forces to support migration. In the right context, however, reduced stress fiber formation and actomyosin could support greater cytoskeletal dynamics to allow for enhanced migration during wound closure as seen in Figure 7A. Next, SKCO15 cells expressing a Rac1 FRET biosensor (31) were used to determine spatiotemporal activation of Rac1, a GTPase known to promote migration through lamella formation and relaxation of contractile forces (29). Unlike RhoA, Rac1 activity was enhanced by exposure to TSP1, which is consistent with our findings of TSP1-mediated enhancement of IEC migration (Figure 8, C and D). To verify these findings, SKCO15 IECs were wounded and exposed to TSP1 for 24 hours before harvest and analysis of active RhoA in pulldown assays with Rhotekin-RBD beads. Data showed significant downregulation of RhoA activation in response to TSP1 compared with untreated control (Figure 8E, *P* < 0.05) and suggest that TSP1 regulates cytoskeletal dynamics through suppression of RhoA activity and promotion of Rac1 activity.

## Discussion

### TSP1 regulates IEC motility and wound healing.

TSP1 is a matricellular protein best characterized as being an important regulator of tissue remodeling and wound repair in the skin. Various studies of epithelial cells of the skin and other tissues such as the corneum demonstrate that TSP1 is critical for migration of epithelial cells in response to injury (32–34). However, the importance of TSP1 during mucosal wound repair in the gut remains poorly understood. In particular, despite its abundant expression in colonic epithelium, very little is known about TSP1 function in IECs compared with other cell types (35–38). This study reveals an important role for epithelially derived TSP1 in the context of intestinal epithelial migration and restitution of mucosal homeostasis in the gut. Specifically, we report that TSP1 increases levels of β1 integrins and active focal adhesions in migrating IECs to promote wound closure in vitro and in vivo. These exciting data emphasize the importance of TSP1 for IEC migratory function during mucosal wound healing.

### Dose-dependent response of TSP1 in IECs.

Although previous studies often characterize TSP1 function at high serum concentrations (>1 μg/mL; 10 nM) (39), dose-response studies performed here reveal that lower plasma-relevant concentrations of 100 ng/mL (~1 nM) TSP1 (40) are optimal for driving epithelial migration and promoting wound repair. In fact, 100 ng/mL TSP1 is considered a physiologically relevant concentration (41), and our data, among others (42), highlight that nanomolar amounts of TSP1 can have profound functional effects, including regulation of IEC migration following intestinal injury. Importantly, we report elevated TSP1 expression in wound-adjacent epithelial crypts that might support IEC migration and re-epithelization. The importance of IEC-derived TSP1 is further highlighted by data showing that mice with IEC-specific knockout of TSP1 exhibit a dramatic defect in colonic mucosal wound healing.

The amount of released TSP1 can greatly vary between acute and chronic inflammation in various diseases (41). Specifically, elevated levels of TSP1 are found in the blood of people with IBD (43, 44). However, more work will need to be done to evaluate how different concentrations of TSP1 affect intestinal epithelial function in vivo. Our findings here suggest that localized increases in TSP1 in colonic epithelium could signal to IECs to affect functional outcomes in conditions such as IBD. That exogenously added TSP1 results in elevated production of TSP1 at the front edge of migrating sheets of IECs highlights the importance of spatiotemporal regulation of TSP1 expression in regulating wound repair. It has been well described in other systems that collectively migrating cells can impose directionality through self-gradients (45, 46). These findings are especially important given that TSP1 was originally described as being both chemoattractive and haptotactic (35–38), suggesting that exogenous TSP1 might disrupt the response of IECs to a gradient of epithelially derived TSP1. Although we do find that TSP1 promotes directionality at high concentrations, more studies would need to be done to determine chemoattractive behavior of IECs in response to exogenous TSP1. As we found dynamic regulation of the 2 Rho GTPases, Rac1 and RhoA, by TSP1, it would be of interest to examine the TSP1-mediated changes in Cdc42 activity, another Rho GTPase that has been found to regulate directionality in various cell types and is important for IEC migration and polarity (47–49).

An interesting previous study examining glioma cell migration and invasion found that apatinib disrupted cytoplasmic interaction between TSP1 and MYH9 (50). These findings suggest an important functional role for endogenous TSP1 production, as exogenously produced TSP1 would likely be excluded from direct access to cytoplasmic myosin. In this context, it may be that mice with IECs deficient in TSP1 would not be “rescued” by the abundant TSP1 produced from infiltrating cells in mucosal wounds in vivo. However, it should be noted that in vitro–attenuated wound closure in TSP1-deficient IECs can be rescued by exogenous TSP1 signaling through surface receptors CD47 and TGF-βR1/2. More work needs to be done to determine the importance of cytoplasmic TSP1-mediated signaling in IECs.

Previous studies suggest that de-adhesion responses mediated by matricellular proteins may lead to a beneficial state for migration termed “intermediate adhesion,” characterized by attenuated actin stress fiber formation with unaltered β1 integrin clustering (2, 51). De-adhesion responses mediated by TSP1 have been reported in studies with fibroblasts and endothelial cells (52–55). In contrast, enhanced adhesion in the presence of TSP1 has been reported for leukocytes (56–58) and several cancer cell lines (59–64). One study described that migrating endothelial cells exhibit biphasic dose-dependent responses to TSP1 while pro-migratory responses of fibroblasts/epithelial tumor cells saturate at high levels of TSP1 (65). Since TSP1-deficient IECs showed increased focal adhesion formation at the leading edge and attenuated wound closure responses, we hypothesize that epithelially expressed TSP1 intrinsically reduces adhesive contacts to enable more effective migration. While TSP1 depletion delayed colonic mucosal wound repair in vivo, addition of high concentrations of TSP1 in vitro were less effective at promoting IEC wound healing. If the response of TSP1 in IECs is biphasic (as has been observed previously in endothelial cells). This suggests that higher concentrations of TSP1 may have negative effects on IEC adhesion dynamics. This hypothesis is supported by a reported association of elevated TSP1 levels with poor clinical outcomes in patients with IBD (43). The importance of TSP1 in promoting intestinal mucosal wound repair, however, is supported by results from other studies (7, 66–70). Although such studies all highlighted the beneficial role of TSP1, the focus was on how TSP1 acts as an antiinflammatory and antiangiogenic signaling protein, and there was no investigation of the effects of TSP1 on epithelial function. Clearly, more studies are needed to better understand the pleiotropic function of TSP1 within different disease contexts given that TSP1 interacts with several receptors expressed by multiple cell types.

### TSP1-mediated signaling through TGF-β1 in IECs.

To better understand the mechanism behind TSP1-dependent regulation of IEC migration, we focused on TGF-β1 signaling, a pathway highly relevant to regulation of intestinal wound repair (71, 72). TGF-β1 has been reported to promote both dedifferentiation of wound-adjacent epithelial cells during mucosal healing (73–78) as well as differentiation and apoptosis of IECs during homeostasis (79). As TSP1 has been shown to activate latent TGF-β1 into its bioactive form (5), we hypothesized that TSP1-induced migration of IECs may be dependent on TGF-β1 signaling. In this study, we report that TSP1-dependent effects on β1 integrin dynamics and IEC migration are dependent on TGF-β1 signaling. Our data also demonstrate a dependency for the TSP1 receptor CD47 in mediating functional effects of TSP1, which led us to examine whether CD47 and TGF-β1 signaling were interdependent. We observed CD47-deficient IECs have attenuated downstream phosphorylation of the TGF-β1 signaling protein SMAD3, which is consistent with a previous report of CD47-deficient IECs exhibiting suppressed TGF-β1 signaling (8). In the absence of CD47, expression of TSP1 and TGF-β1–related signaling proteins was not upregulated following TGF-β1 treatment, suggesting that CD47-dependent transcriptional mechanisms may be driving these responses. Interestingly, TSP1 expression localized at the luminal surface of healthy murine colonic crypts containing fully differentiated epithelial cells. Regulated expression of TSP1 (and therefore TGF-β1 signaling) at the top of epithelial crypts may thus be important for limiting detrimental dedifferentiation through TGF-β1 at the base of colonic crypts during homeostasis (76, 80) and provides an important future direction of study. As SMAD3 and other TGF-β1 signaling genes have been linked to the pathobiology of IBD through genome-wide association studies and other genomic screenings (81–84), understanding molecular mechanisms whereby TSP1 activates TGF-β1 will likely provide a greater understanding of IBD pathogenesis.

Of note, integrins have been previously demonstrated to be activators of TGF-β1 (85, 86). As we show an increase in β1 integrin expression following TSP1 treatment in IECs, it stands to reason that upregulation of integrins might directly promote TGF-β1 signaling during mucosal repair. In keeping with this, previous studies demonstrated that CD47-deficient epithelial cells, which do not respond to TSP1 treatment, have reduced TGF-β1 signaling concomitant with reduced β1 integrins (8). Comparing the contributions of integrin-mediated activation of TGF-β1 to activation by TSP1 would be an interesting direction for future studies.

### Context-dependent functions of TSP1.

In this report, we show that mice deficient in TSP1 have delayed intestinal mucosal healing responses. In keeping with this observation, there are previous reports of defective dermal wound healing in mice deficient in TSP1 (4, 6). Additionally, previous work has demonstrated that IEC-specific knockdown of CD47 (a receptor for TSP1) results in impaired colonic wound repair (8). However, CD47-deficient dermal wounds are characterized by elevated expression of TSP1 and enhanced wound repair (6). These studies highlight context- and tissue-specific regulation of TSP1/CD47 signaling during wound repair. It is evident that the lack of CD47 expression in dermal wounds does not prevent TSP1 from binding to other receptors, including integrins and latent TGF-β1, to promote wound repair (6). In contrast with this, we report that TSP1-dependent effects on IEC migration are interdependent on both CD47 and TGF-β1. TSP1-dependent signaling pathways are therefore clearly cell specific, and studies such as in this report were necessary to better understand the functional role(s) of TSP1 in the context of colonic mucosal wound healing.

From multiple other reports, it is clear that TSP1 function in vivo likely differs significantly depending on the cellular systems being studied. Given the ubiquitous nature of TSP1 and TGF-β1 and expression by many cell types including platelets, fibroblasts, macrophages, and endothelial cells, as well as its reported roles in diverse processes including aging, wound healing, cancer, and angiogenesis (87), tissue-targeted transgenic approaches are needed to better understand functions of TSP1 during disease. As an important example, both immune and endothelial cell–derived TSP1 have been shown to play functionally distinct roles in vascular remodeling (88). Similarly, in our study, we used IEC-specific knockdown of TSP1 as well as bone marrow chimera models to elucidate the contribution of hematopoietic versus epithelial cell–produced TSP1 during colonic repair. From our studies, it is clear that loss of TSP1 attenuates colonic wound healing in vivo and highlights for the first time to our knowledge that TSP1 derived specifically from the intestinal epithelium plays a vital role in driving mucosal repair in the gut.

### Conclusion.

In summary, we report physiologically relevant concentrations of TSP1 promote epithelial migration through TGF-β1 signaling and regulation of focal adhesion dynamics. We show that IECs represent an important source of TSP1 during mucosal repair in the gut. Given the pleiotropic effects of TSP1 that have been reported, future work is needed to better define molecular mechanism(s) by which TSP1 promotes mucosal wound repair in different tissues. Increased understanding of how TSP1 regulates epithelial repair has the potential to inform rational therapies that promote resolution of chronic injury/inflammation in mucosal tissues such as the gut.

## Methods

### Sex as a biological variable.

Our study examined male and female animals, and similar findings are reported for both sexes.

### Antibodies and reagents.

Anti–integrin β1 (clone KMI6; ab95623; Western blot [WB]: 1:500) and anti–thrombospondin-1 (ab85762; immunofluorescence [IF]: 1:100, WB: 1:500) antibodies were purchased from Abcam. Anti–phospho-p130CAS (Tyr410; 4011; WB: 1:1,000), anti-p130CAS (E1L9H; 13846; WB: 1:1,000), anti-pMLC (Ser19; 3671; WB: 1:500), anti–phospho-Smad3 (Ser423/425 clone C25A9; 9520; WB: 1:500), and anti-Smad2/3 (catalog 5678; WB: 1:1,000) antibodies were purchased from Cell Signaling Technology. Goat anti-mouse E-Cadherin (AF748; IF: 1:100) antibodies were purchased from R&D Systems, Bio-Techne. Anti-GAPDH (G9545; WB: 1:20,000) antibody was purchased from MilliporeSigma. Alexa Fluor 488– and 647–conjugated secondary antibodies (IF: 1:1,000) were purchased from Thermo Fisher Scientific. Secondary antibodies for WB analysis were obtained from Jackson ImmunoResearch Laboratories. Recombinant human TGF-β1 (catalog 616455) was purchased from CalBioChem, and recombinant mouse (7859-TH-05) and human TSP1 (03074-TH-050) were purchased from R&D Systems, Bio-Techne. Purity of recombinant mouse TSP1 was tested for contaminants by tandem mass spectrometry and revealed no evidence of contaminating TGF-β peptides. TGF-β1 inhibitors SIS3 (catalog 15945) and ITD1 (catalog 23326) were purchased from Cayman Chemical and used at a concentration of 10 μM. Human IECs, T84 and SKCO15, were cultured as previously described (89). SKCO15 were provided and authenticated by Enrique Rodriguez-Boulan (Weill Cornell Medical College, New York, New York, USA). T84 cells were obtained from ATCC (CCL-248).

### Mice.

*Cd47*-knockout mice on a C57BL/6 background were purchased from The Jackson Laboratory and bred in-house at the University of Michigan in Ann Arbor. *Thbs1*^–/–^ mice were also bred in-house on a C57BL/6 background (90). Thrombospondin-1–floxed mice were gifted by Nader Sheibani (University of Wisconsin-Madison, Madison, Wisconsin, USA) (91). Mice were housed under specific pathogen–free conditions and used at 3–4 months of age.

### Primary murine colonoid culture.

Murine colonic epithelial colonoids were isolated and maintained in culture as described previously (8). Briefly, the large intestine was dissected and flushed with phosphate-buffered saline (PBS) and transferred to chelation buffer (50 mM EDTA, PBS) for 40 minutes. The intestine was shaken to remove crypt cells and then incubated in PBS with 43 mM sucrose and 55 mM sorbitol. For 3D colonoids, isolated intestinal crypts from mice were embedded in Matrigel (BD Biosciences) (30 μL/well) and maintained in LWRN-conditioned complete media supplemented with 50 ng/mL recombinant human EGF (R&D Systems, Bio-Techne) and 100× antibiotics-antimycotic (Corning). To generate 2D epithelial intestinal monolayers from murine 3D colonoids, a single-cell suspension was obtained by resuspension in 0.05% Trypsin/0.5 mM EDTA. Trypsin was inactivated by adding 1 mL advanced DMEM/F12-containing 10% FBS. Dissociated cells were passed through a 40 μm cell strainer, then cultured on collagen type IV–coated, 48-well tissue culture plates (both from Corning) until confluence was achieved (~48 hours). Murine 2D cultures were maintained in LWRN complete media supplemented with 50 ng/mL recombinant human EGF and antibiotics-antimycotic. For ex vivo monolayers, isolated intestinal crypts from mice were directly cultured on collagen type IV–coated 48-well tissue culture plates until confluence was achieved (48–72 hours) (92).

### IEC migration assay.

Chemokinetic migration of ex vivo–isolated colonoids in response to exogenous TSP1 on collagen IV was tracked using a Zeiss Axio Observer microscope with motility tracked every 30 minutes for 24 hours. Motility of individual epithelial leader cells within discrete clusters was tracked using ImageJ Manual Tracking (NIH) and analyzed using the Microsoft Excel–based algorithm DiPer (12).

### Adhesion array assay.

Effects of TSP1 on adhesion of murine ex vivo–isolated colonoids were determined using ECM Cell Adhesion Array Kits (MilliporeSigma) according to manufacturer protocol with minor changes. Colonoids isolated from mice were counted and plated in the appropriate strips at 800 crypts per well. Colonoid-derived IECs were then cultured in LWRN complete media with 50 ng/mL recombinant human EGF for 18 hours at 37°C and 5% CO_2_ to allow attachment. After attachment, cells were gently washed 3 times, lysed, and labeled with CyQuant GR dye. Fluorescence was read at excitation/emission 485/530 nm using a BioTek Cytation 5 fluorescence plate reader.

### Spatiotemporal dynamics of Rac1 and RhoA activation.

To study activation of Rac1 and RhoA in migrating epithelial monolayers, we employed single-chain biosensors previously described (30, 31, 93) to detect the activation of Rac1 (pLenti-Rac1-2G, Addgene plasmid 66111) and RhoA (pLentiRhoA2G, Addgene plasmid 40179). Rac1 and RhoA biosensors consist of a Rac1- or RhoA-binding domain of the effectors p21 activated kinase and Rhotekin, respectively, that bind to GTP-Rac1 or GTP-RhoA. These constructs are followed by TFP, a linker of optimized length, a pH-insensitive yellow fluorescent protein variant, and either full-length Rac1 or RhoA. Lentivirus particles containing the respective plasmids were generated by the University of Michigan Medical School Vector Core. To establish cell lines expressing Rac1 or RhoA biosensors, we used the spinfection protocol (1,200*g* for 30 minutes at room temperature) to induce the lentiviral transduction of either pLenti-Rac1-2G or pLentiRhoA2G constructs in 30%–40% confluent SKCO15 cells. Cells were puromycin-selected during 2 subcultures and maintained at the same concentration (1 μg/mL) to ensure proper expression.

To study Rac1 and RhoA activation patterns during directed cell migration, transduced SKCO15 cells were plated at a density of 1 × 10^5^ cells on collagen IV–coated #1.5 polymer coverslip (Ibidi μ-slide Chemotaxis with collagen IV, catalog 80326). After 24 hours, cells were exposed to 100 ng/mL of TSP1 administrated through a single injection port for varying periods of time. The activation patterns of Rac1 and RhoA were then examined by measuring the FRET/TFP ratio in cell islands whose lamellae were facing the TSP1-containing reservoir. Images of the cells were taken using a Leica SP8 confocal microscope with fluorescence lifetime imaging. Images of the cells were taken at 0-, 2-, and 24-hour time points after TSP1 treatment. Each image was captured every 5 seconds for 15 minutes, with a total of 180 images for each corresponding time point. TFP and FRET images from Rac1 and RhoA biosensors were excited at 460 nm, and TFP and FRET emission spectra were recorded at 480–512 and 520–567 nm, respectively. Finally, the TFP and FRET images were processed and quantified using ImageJ, following a previously published method (94).

### Wound healing assays.

For in vitro experiments, 2D cultures of murine epithelial colonoids or human epithelial cell lines were subjected to scratch wounding as described previously (8). Monolayers were cultured on collagen type IV–coated (C5533, MilliporeSigma), 48-well tissue culture plates until reaching confluence and monolayers injured using a 10 μL pipette tip under suction. Medium was changed after wounding and video quantification of scratch-wound closure was performed by imaging wounds at 60-minute intervals using an Axiovert Observer live cell microscopy system (Zeiss). Wound closure was quantified at the indicated time points using ImageJ software, calculated as percentage reduction of cell-free surface area compared with immediately after wounding (time = 0 hours). Wound closure was then normalized to average wound closure for WT control or untreated control within each independent experiment. For Western blot analysis, wounds were created in a grid pattern to enrich the fraction of migratory IECs and samples harvested at indicated times.

For in vivo wounding experiments, a biopsy-based mucosal wound model was employed using a high-resolution video endoscope (Coloview Veterinary Endoscope, Karl Storz) equipped with biopsy forceps to create biopsy-induced injury of the colonic mucosa at 5 to 7 sites along the dorsal aspect of the colon of anesthetized mice (i.p. injection of ketamine 100 mg/kg, xylazine 5 mg/kg). Wound healing was quantified at 24 hours and 72 hours after injury. Endoscopic procedures were viewed with high-resolution (1,024 × 768 pixels) images on a flat-panel color monitor. Each wound region was digitally photographed at 24 hours and 72 hours, and wound areas were calculated using ImageJ. In each experiment, 3 to 5 wounds per mouse were analyzed by individuals masked to sample groups.

### RNA extraction and qPCR.

The mRNA expression levels of various genes were measured in human and mouse samples as described previously (95). In brief, total RNA was extracted from the samples using the RNeasy Mini Kit (catalog 74106, QIAGEN) according to the manufacturer’s instructions. Reverse transcription was performed using the iScript Reverse Transcription Supermix for RT-qPCR (catalog 1708840, Bio-Rad). qPCR amplification was then performed using the iQ SYBR Green Supermix (catalog 1708880, Bio-Rad) in a CFX Connect Real-Time PCR Detection System (Bio-Rad). Target mRNA levels were normalized to those of *Rps18* or *Gapdh* as the internal control in each sample and calculated by the 2-ΔΔCt method. The results are expressed as ratios relative to the average for the control group. The following primer pairs were used: *Mus musculus* *Cd47*, (forward) 5′-TGCGAAGTGACAGAGTTATCC-3′ and (reverse) 5′-TCATTTGGAGAAAACCACGAAAC-3′; *Mus musculus* *Thbs1*, (forward) 5′-CAACCGCATTCCAGAGTCTG-3′ and (reverse) 5′-GCCAGTGTTGTCTTTCCGTT-3′; *Mus musculus* *Tgfbr1*, (forward) 5′-TGCTCCAAACCACAGAGTAGGC–3′ and (reverse) 5′-CCCAGAACACTAAGCCCATTGC-3′; *Mus musculus* *Tgfbr2*, (forward) 5′-CCTACTCTGTCTGTGGATGACC-3′ and (reverse) 5′-GACATCCGTCTGCTTGAACGAC-3′; *Mus musculus* *Tgfb1*, (forward) 5′-GAAGGACCTGGGTTGGAAGT-3′ and (reverse) 5′-CCGGGTTGTGTTGGTTGTAG-3′; *Mus musculus* *Rps18*, (forward) 5′-ACTTTTGGGGCCTTCGTGTC-3′ and (reverse) 5′-GCCCAGAGACTCATTTCTTCTTG-3′; *Mus musculus* *Gapdh*, (forward) 5′- ACACATTGGGGGTAGGAACA-3′ and (reverse) 5′-AACTTTGGCATTGTGGAAGG-3′.

### DSS-induced colitis.

Mice were provided with 3% w/v DSS (40 kDa, 14489, Affymetrix) in drinking water ad libitum for indicated times followed by 3–5 days with normal drinking water (96). Clinical disease assessment was obtained daily, with scores of 0–4 assigned for weight loss, stool consistency, and presence of blood in stools. Individual scores were added, and the average was recorded as the DAI where higher DAI values reflect increasing severity of colitis. At the end of the experiment, at either day 9 or day 11 after DSS treatment, mice were sacrificed, and colons were harvested. H&E staining of sections of Swiss roll mounts of the entire colon (8 μm thick) was performed to quantify colonic mucosal injury. Percentage of injury/ulceration was calculated as a ratio of the length of injured/ulcerated area (≥50% crypt loss) relative to the entire colon length as described previously (97).

### Immunofluorescence and RNAscope.

2D colonoid monolayers were grown on plastic chamber slides (Thermo Fisher Scientific, 177445) and fixed with 4% paraformaldehyde (PFA). PFA-fixed monolayers were permeabilized with 0.5% Triton X-100 for 10 minutes. Monolayers were blocked with 3% goat or donkey serum in Dulbecco’s PBS with 0.05% Tween 20 blocking buffer for 30 minutes. Primary antibodies were diluted in blocking buffer, and cells were incubated overnight at 4°C. Cells were washed with PBS with 0.05% Tween 20, and fluorescently labeled secondary antibodies or phalloidin was diluted in blocking buffer followed by incubation for 1 hour at room temperature. Cells were washed and mounted in Prolong Gold antifade agent (Thermo Fisher Scientific, P36930). Frozen colonic sections (6–8 μm) were fixed with 4% PFA, and immunolabeling was performed as described above. Fluorescence imaging was performed with a Nikon A1 confocal microscope in the Microscopy & Image Analysis Laboratory Core at the University of Michigan and analyzed using ImageJ. To quantify focal adhesions containing phospho-p130Cas, fluorescence of phospho-p130Cas outside of leader cells (region of interest, ROI) was removed from images. Images were then converted to 8-bit. Base exponent of all pixel values was computed, then converted using Laplacian of Gaussian filter. Images were then processed using Threshold, and focal adhesions were automatically counted using Analyze Particles. To quantify fluorescence intensity of F-actin and pMLC, the ROI was drawn around migrating epithelial monolayers and measured for integrated density, subtracted for background, and normalized to untreated WT control in each independent experiment.

Localization of mouse *Thbs1* mRNA was determined using 8 μm sections of formalin-fixed and ethanol-dehydrated tissues. Following incubation with hydrogen peroxide (ACDbio, catalog 322000), tissues were digested by protease plus treatment (catalog 322330). *Thbs1* probe for mouse (ACDbio, catalog 457898) was hybridized for 2 hours followed by signal amplification. Chromogenic detection was performed via RNAscope 2.5 HD Detection kit-RED (ACD, catalog 322350).

### Western blot.

Cells were lysed in RIPA buffer (150 mM NaCl, 1% NP-40, 0.5% deoxycholic acid, 0.1% SDS, 50 mM Tris pH 8.0) with protein and phosphatase inhibitors, followed by sonication and centrifugation. Protein concentrations were quantified using the Bio-Rad DC Protein Assay. Equal quantities of protein samples were loaded into 10% polyacrylamide gels for SDS-PAGE and transferred to 0.2 μm nitrocellulose membrane (Bio-Rad). Membranes were blocked with either 5% milk powder/TBS/Tween 20 or 3% BSA/TBS/Tween 20 for phosphorylated proteins, followed by overnight incubation with primary antibodies at 4°C. Membranes were then incubated with appropriate HRP-conjugated secondary antibodies (Jackson ImmunoResearch Laboratories catalog 111-035-144) for 1 hour at room temperature, followed by development using Western ECL Substrate, with images captured via ChemiDoc imager (Bio-Rad). For phosphorylated SMAD3 quantification, the lower of the 2 bands was quantified (52 kDa), as done by Jeon et al. (98). The upper band is likely phosphorylated SMAD2 because its observed molecular weight is 58 kDa (99) and because it is phosphorylated at the SSXS motif at the C-terminus, similar to SMAD3 (100). For phosphorylation of p130Cas, the upper band of the 2 bands appears more prominently 24 hours after injury and corresponds to an isoform of p130Cas phosphorylated at multiple sites (101).

For RhoA activation, 90 μg of protein lysate was mixed with GST-Rhotekin beads (Cytoskeleton Inc, catalog RT102-A) according to the manufacturer’s protocol. Total lysate and active RhoA pulldown samples were then immunoblotted using standard methods above.

### Bone marrow chimera generation.

For total bone marrow (BM) transplant experiments, donor BM cells were harvested from WT mice (C57BL/6 CD45.1; 002014, The Jackson Laboratory) and *Thbs1^–/–^* (CD45.2) mice. Recipient mice were irradiated twice at 5 Gy with a 4-hour interval in between. The irradiated mice received 5 × 10^6^ donor BM cells via retroorbital sinus injection in 200 mL of PBS. Blood samples were collected by facial vein bleed from the recipients 4 and 8 weeks after BM transplantation to confirm engraftment. Experiments using the recipients were conducted 8–10 weeks after BM transplantation.

### Statistics.

All data are derived from 2 or 3 independent experiments. Statistical analysis was performed with GraphPad Prism software. Parametric or nonparametric tests were performed to evaluate statistical significance after finding if datasets were normally distributed. Unpaired, 2-tailed Student’s *t* test was used to compare 2 independent groups. One-way or 2-way ANOVA with indicated multiple comparisons test were used to compare multiple groups with 1 or 2 independent variables, respectively. Differences were considered significant when *P* < 0.05. Results are expressed as means ± SEM when not displayed as a box-and-whisker plot.

### Study approval.

All experiments were approved and conducted in accordance with guidelines set by the University of Michigan Institutional Animal Care and Use Committee.

### Data availability.

The authors declare that all data supporting the findings of this study are available within the paper and its supplement files, including the Supporting Data Values XLS file, and from the corresponding author on reasonable request.

## Author contributions

ZSW planned and was involved in all experiments as well as wrote the manuscript. MQ and JM were involved in the in vivo colonoscopy model. ARS helped plan and execute the experiments with Rac1 and Rho biosensors. SF helped plan and execute experiments with Rhotekin bead pulldown. MQ and VGH helped plan and execute experiments with in situ hybridization (RNAscope). QL and CHK helped generate BM chimeras. JCB, KDH, AN, and CAP helped plan and develop the manuscript.

## Supplementary Material

Supplemental data

Unedited blot and gel images

Supporting data values

## Version 1 07/30/2024

In-Press Preview

## Version 2 09/10/2024

Electronic publication
