# Supplementary material for: Critical role of thrombospondin-1 in promoting intestinal mucosal wound repair
Source: JCI Insight. 2024 Jul 30;9(17):e180608. doi: 10.1172/jci.insight.180608 (PMC11385097; doi:10.1172/jci.insight.180608)
Supplement: Supplemental data [file jciinsight-9-180608-s131.pdf]

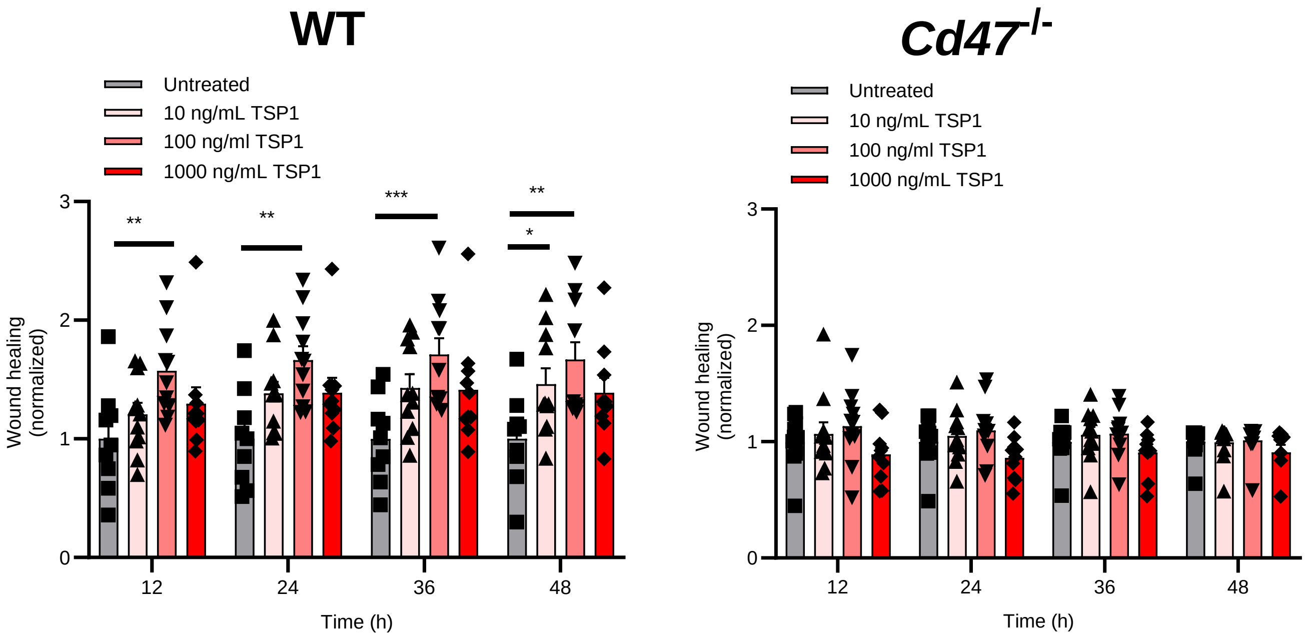

Supplemental Figure 1: Migration of WT and CD47-deficient IECs after TSP1 treatment.
Wound closure following scratch wounding for WT and CD47-deficient mouse IECs in response to thrombospondin-1 over 48 hours (n=10, N=2 independent experiments). Data shown as normalized to untreated conditions at each timepoint. Performed two-way ANOVA with Dunnett's multiple comparison test. * $p<0.05$, ** $p<0.01$, $p<0.001$. Mean +/- SEM.

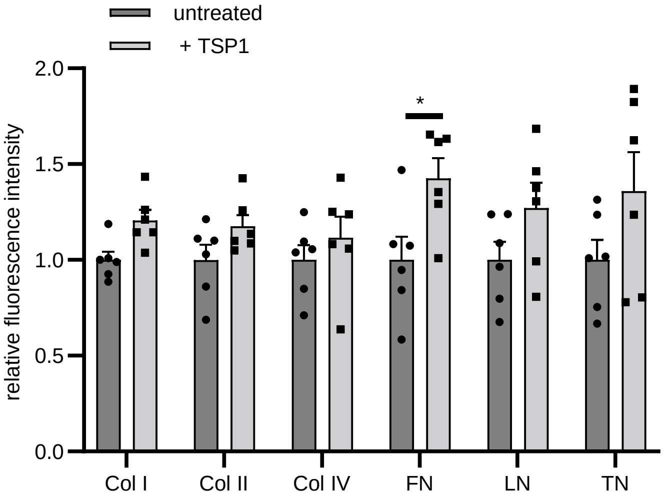

Supplemental Figure 2: Adhesion of IECs to different ECM substrates after TSP1 treatment.
Attachment of murine IECs to various extracellular matrices after 18h TSP1 treatment as measured after fluorescent labeling of lysate (n=6, N=2 independent experiments). Abbreviations: Collagen I (Col I), Collagen II (Col II), Collagen IV (Col IV), Fibronectin (FN), Laminin (LN), Tenascin (TN). No adhesion to Vitronectin was detected. Performed two-way ANOVA with Šídák multiple comparison test. * $p<0.05$. Mean +/- SEM.

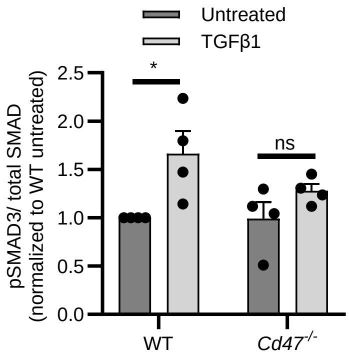

Supplemental Figure 3: Thrombospondin-1 expression in WT and CD47-deficient IECs after TSP1 treatment.
Quantification of immunoblot of pSMAD3 and SMAD2/3 in WT and *Cd47*$^{-/-}$ murine IECs treated with TGFB1 (2 ng/mL) 24 hours post-wounding (N=4 independent experiments). Performed two-way ANOVA with Šídák multiple comparison test. * $p<0.05$, ns = nonsignificant. Mean +/- SEM.

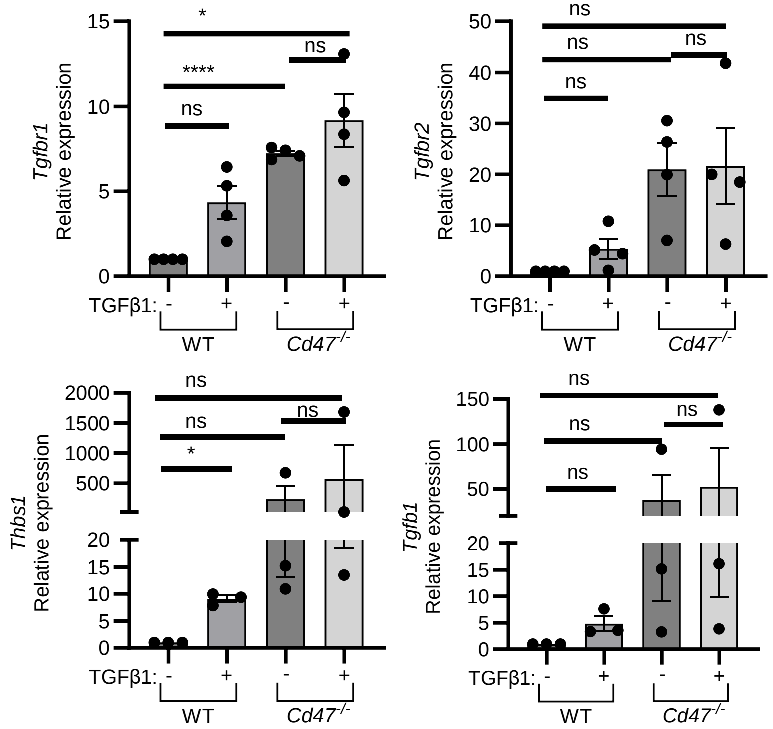

Supplemental Figure 4: Upregulated mRNAs in WT and CD47-deficient IECs after TSP1 treatment.
qPCR of mRNA in WT and *Cd47*$^{-/-}$ murine IECs treated TGFB1 (2 ng/mL) 4 hours post-wounding normalized to WT untreated (N=4 independent experiments). Performed one-way ANOVA with Tukey's multiple comparison test. * p<0.05, ns = nonsignificant. Mean +/- SEM.

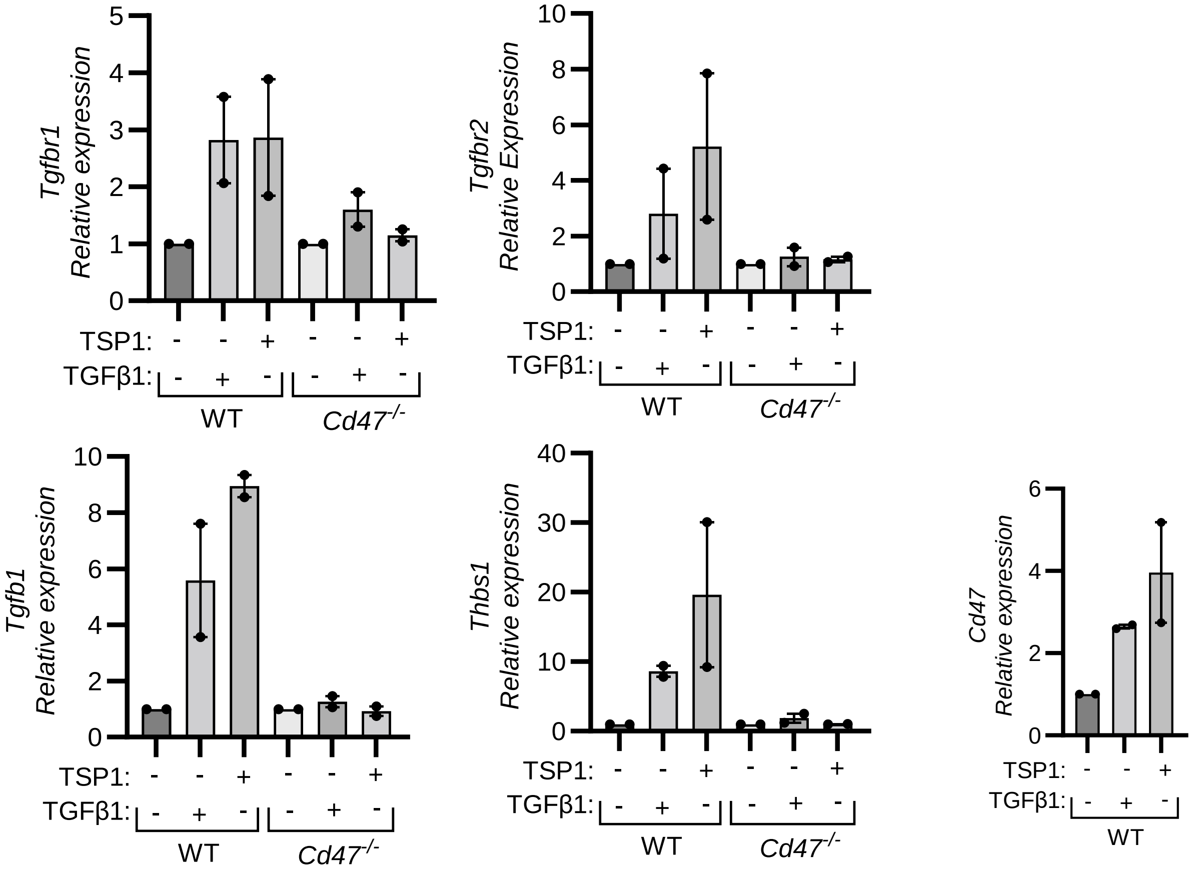

Supplemental Figure 5: Thrombospondin-1 also promotes the expression of TGFβ-related genes
qPCR of mRNA expression relative to untreated condition in WT and *Cd47*$^{-/-}$ murine IECs treated with TGFβ1 (2 ng/mL) or TSP1 (100 ng/mL) 4 hours post-wounding (N=2 independent experiments). Mean +/- SEM.
