# Supplementary material for: Critical role of thrombospondin-1 in promoting intestinal mucosal wound repair
Source: JCI Insight. 2024 Jul 30;9(17):e180608. doi: 10.1172/jci.insight.180608 (PMC11385097; doi:10.1172/jci.insight.180608)

Full unedited blots/gels

Full unedited blots/gels for Figure 3B

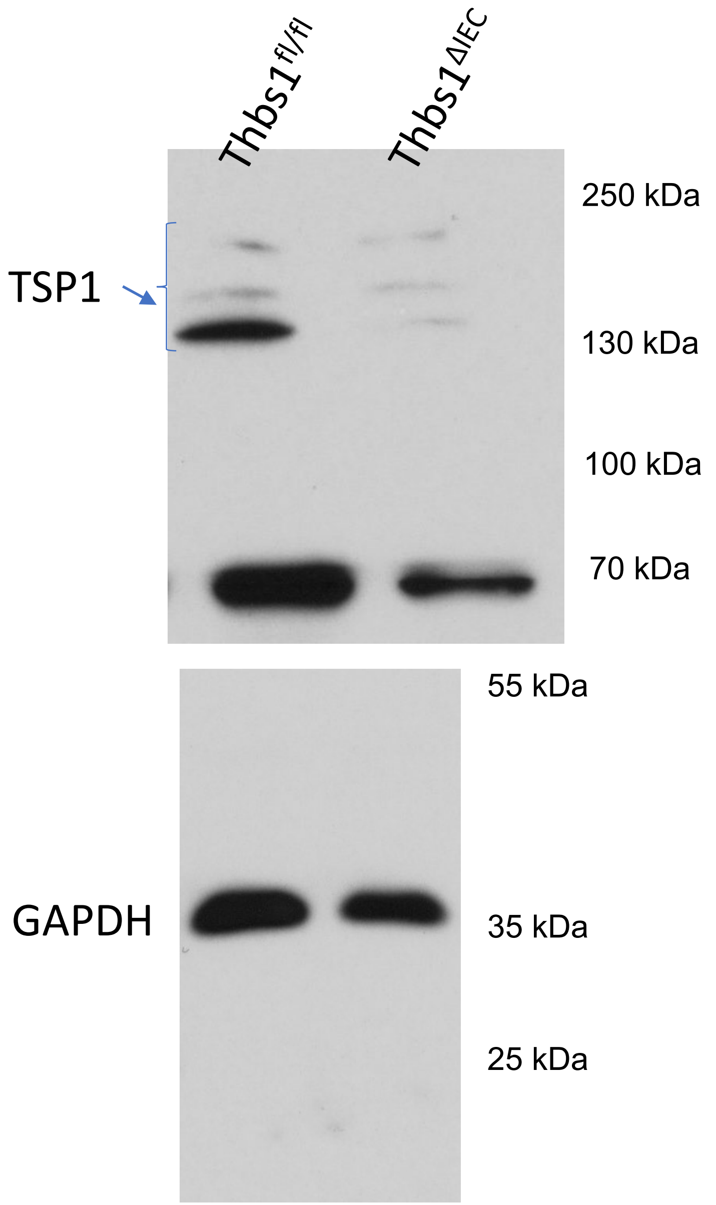

Full unedited blots/gels for Figure 5C

2023-07-10

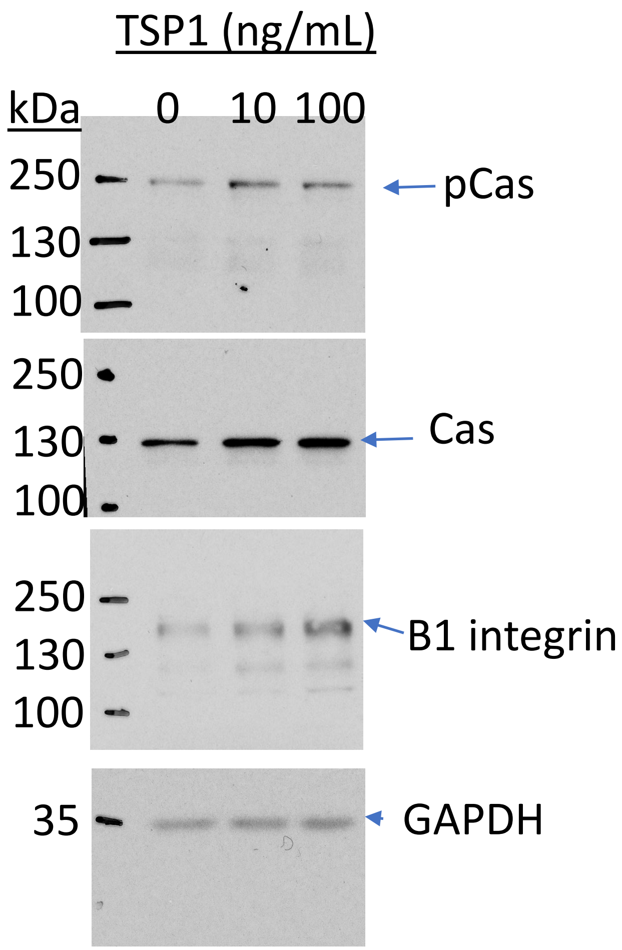

Full unedited blots/gels for Figure 6A

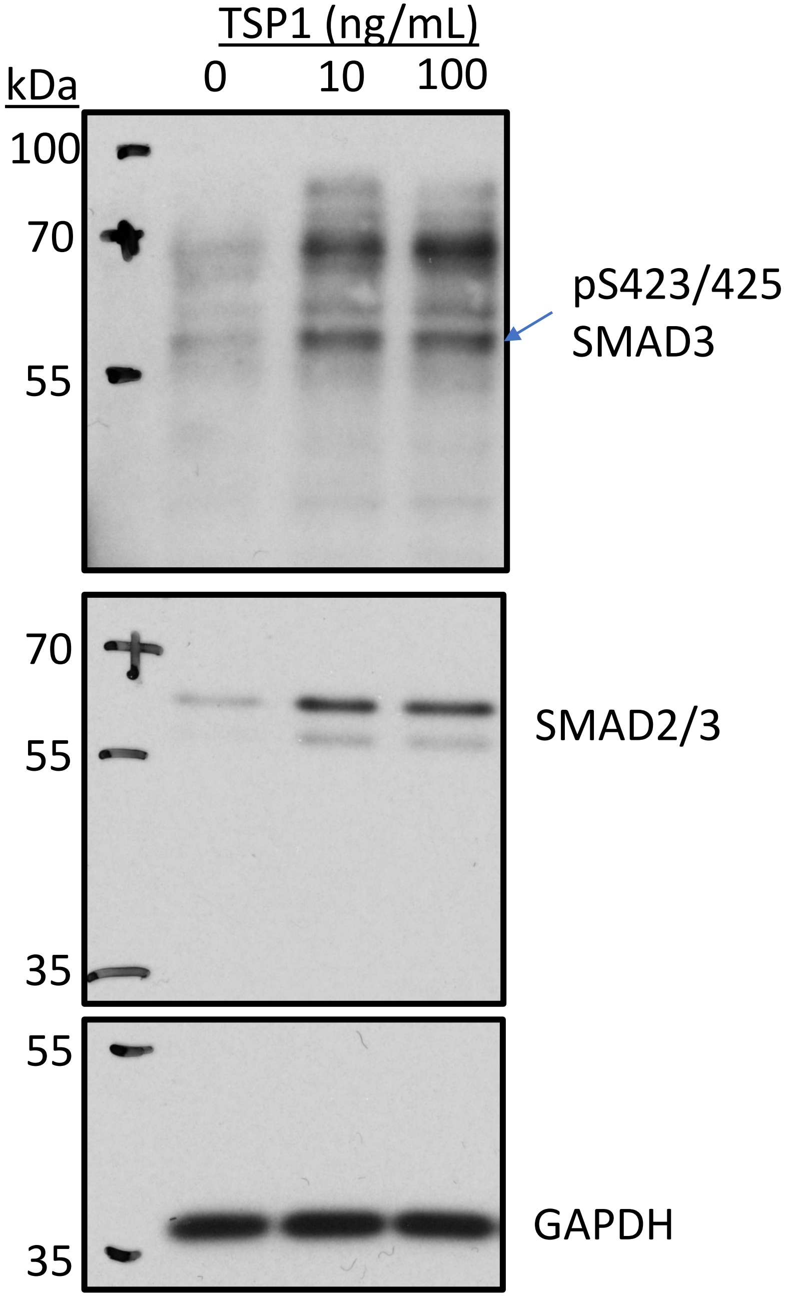

Full unedited blots/gels for Figure 6B

2020-07-30

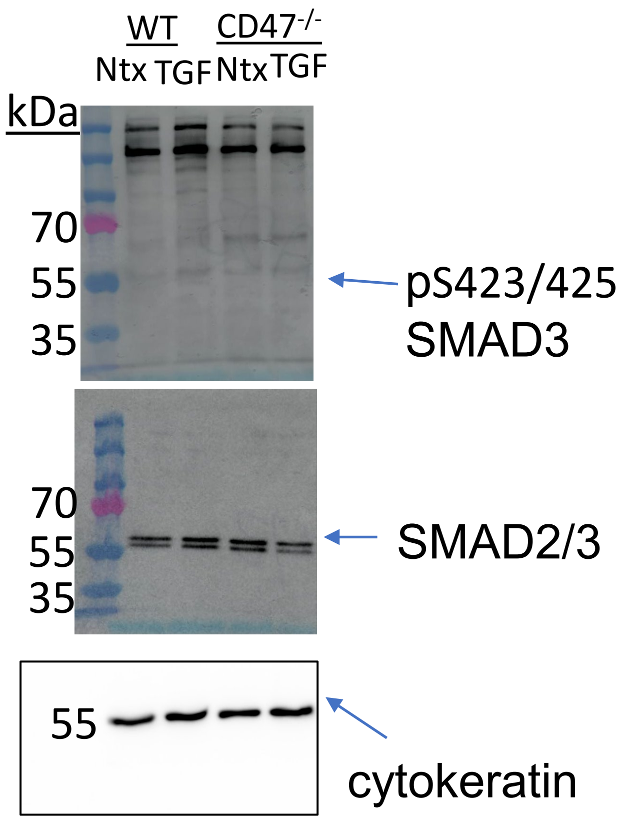

Full unedited blots/gels for Figure 6E

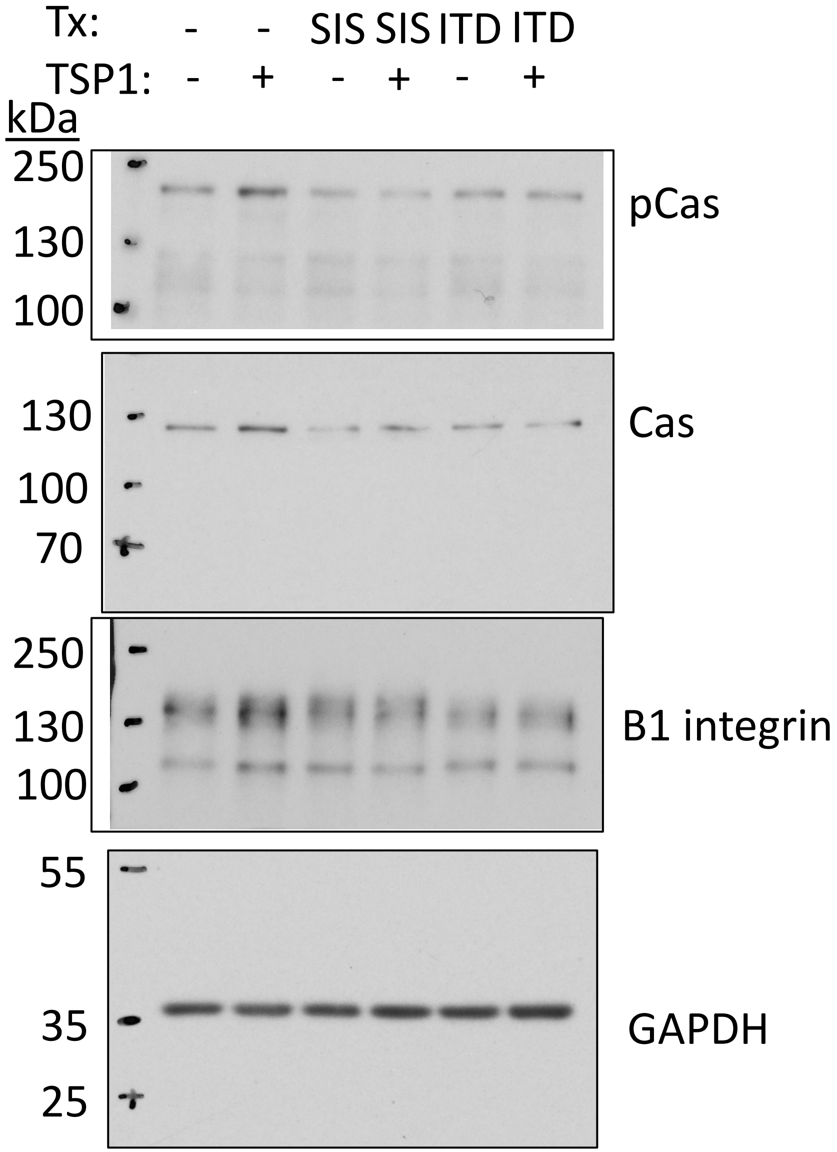

Full unedited blots/gels for Figure 8E

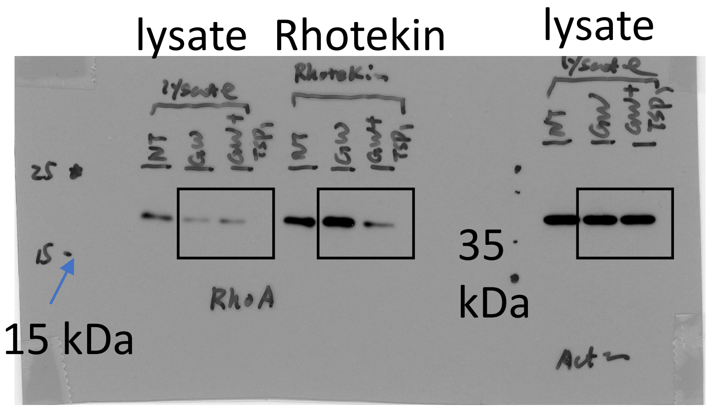

Supplement: Unedited blot and gel images [file jciinsight-9-180608-s132.pdf]
